# 3-Pyridylnitrene, 2- and 4-pyrimidinylcarbenes, 3-quinolylnitrenes, and 4-quinazolinylcarbenes. Interconversion, ring expansion to diazacycloheptatetraenes, ring opening to nitrile ylides, and ring contraction to cyanopyrroles and cyanoindoles

**DOI:** 10.3762/bjoc.9.84

**Published:** 2013-04-17

**Authors:** Curt Wentrup, Nguyen Mong Lan, Adelheid Lukosch, Pawel Bednarek, David Kvaskoff

**Affiliations:** 1 School of Chemistry and Molecular Biosciences, The University of Queensland, Brisbane, Qld 4072, Australia; 2 Institut de Chimie Organique, Université de Lausanne, CH-1009 Lausanne, Switzerland; 3 Fachbereich Chemie der Universität Marburg, D-3550 Marburg, Germany

**Keywords:** carbene-nitrene interconversion, diazepines, flash vacuum thermolysis, matrix photochemistry, nitrile ylides, reactive intermediates

## Abstract

Precursors of 3-pyridylnitrene and 2- and 4-pyrimidinylcarbenes all afford mixtures of 2- and 3-cyanopyrroles on flash vacuum thermolysis, but 3-cyanopyrroles are the first-formed products. 3-Quinolylnitrenes and 4-quinazolinylcarbenes similarly afford 3-cyanoindoles. 2-Pyrimidinylcarbenes rearrange to 3-pyridylnitrenes, but 4-pyrimidinylcarbenes and 4-quinazolinylcarbenes do not necessarily rearrange to the corresponding 3-pyridylnitrenes or 3-quinolylnitrenes. The ring contraction reactions are interpreted in terms of ring opening of either the nitrenes or the diazacycloheptatetraenes to nitrile ylides.

## Introduction

**Scheme 1:**
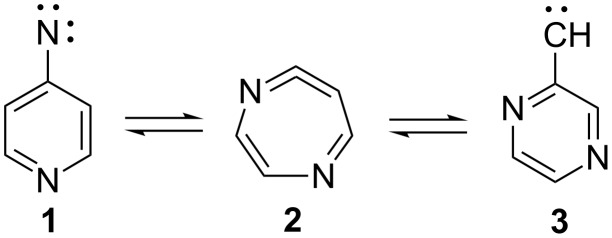
4-Pyridylnitrene–2-pyrazinylcarbene interconversion.

**Scheme 2:**
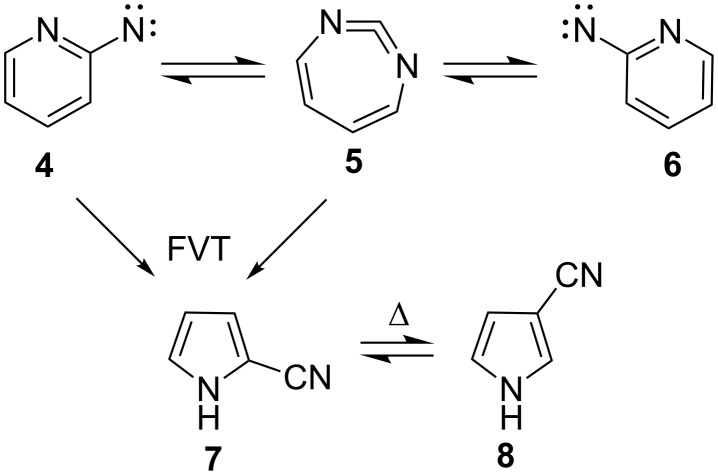
Ring expansion and ring contraction in 2-pyridylnitrene (**4**).

A multitude of rearrangements of heterocyclic nitrenes have been described in a recent review [1] and in several books [2–5]. This is illustrated by the ring expansion of 4-pyridylnitrene (**1**) and 2-pyrazinylcarbene (**3**) to 1,5-diazacyclohepta-1,2,4,6-tetraene (**2**, Scheme 1) [6]. Similarly, 2-pyridylnitrene (**4**) interconverts with 1,3-diazacyclohepta-1,2,4,6-tetraene (**5**) under conditions of photolysis in solution or in matrices as well as under flash vacuum thermolysis (FVT) (Scheme 2) [1,7–10].

The end products under FVT conditions are the 2- and 3-cyanopyrroles **7** and **8** [10–11]. A competing ring-opening of 2-pyridylnitrenes to cyanobutadienylnitrenes can lead to the formation of glutacononitriles in low yields [12].

**Scheme 3:**
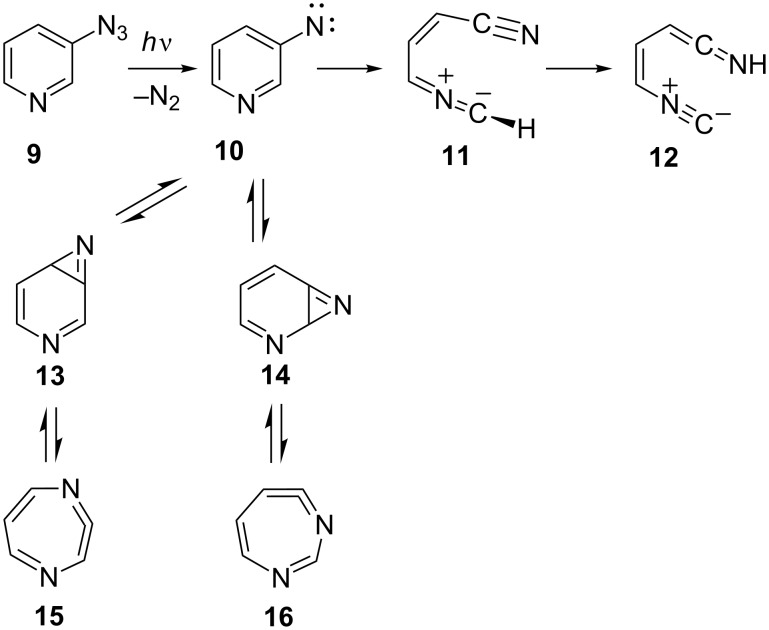
Ring opening and ring expansion in 3-pyridylnitrene (**10**).

In contrast, 3-pyridylnitrene (**10**) undergoes a different type of ring opening to the observable nitrile ylide **11** and subsequently the ketenimine **12** (Scheme 3) [13]. Nitrile imines [14] and nitrile ylides [15–16] may have either allenic or propargylic structures, and for this reason their cumulene-type IR absorptions can occur over a wide frequency range, 1900–2300 cm^−1^, depending on substituents. The IR absorption of **11** was observed at 1961 cm^−1^, indicating an allenic structure. Nitrene **10** can also undergo ring expansion to two diazacycloheptatetraenes **15** and **16** via the azirenes **13** and **14** (Scheme 3) [13]. The diazacycloheptatetraenes were not observed directly in this study, but aza- and diazacycloheptatetraenes have been observed in several other cases [1,17–18] and the ring-expansion reactions have been the subject of detailed theoretical investigation [19].

## Results and Discussion

### 3-Pyridylnitrene

**Scheme 4:**
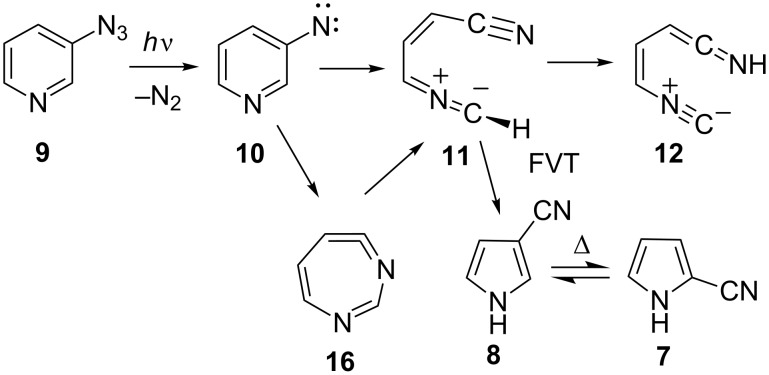
Ring opening and ring contraction in 3-pyridylnitrene (**10**) and diazacycloheptatetraene **16**.

**Figure 1:**
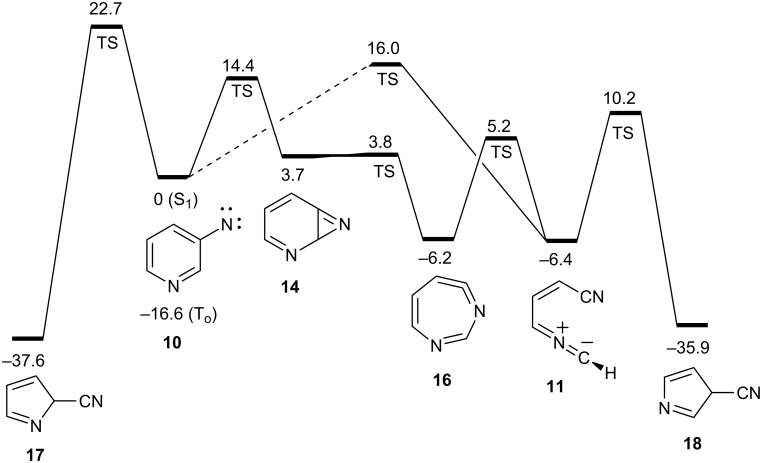
Energy profile for the ring opening and ring contraction in 3-pyridylnitrene **10** and 1,6-diazacyclohepta-1,2,4,6-tetraene (**16**, energies in kcal/mol at the B3LYP/6-31G* level).

Flash vacuum thermolysis (FVT) of 3-azidopyridine (**9**) yields predominantly 3-cyanopyrrole (**8**, Scheme 4). Under the mildest conditions, FVT of **9** at 370 °C/10^−3^ mbar generates **8** and **7** in a 3:1 ratio. A temperature increase to 500 °C causes the ratio to drop to 1.2:1 due to the thermal interconversion of **7** and **8** [10,20]. The reaction mechanism was probed by calculations at the B3LYP/6-31G* level, which has been found to be adequate for other, similar reactions [11,13,17,21]. The transition state for the ring opening of the cyclic ketenimine **16** to the (s,*Z*)-nitrile ylide **11** lies 5 kcal/mol above the open shell singlet nitrene S_1_ **10**, i.e., the barrier is only 11 kcal/mol above the cyclic ketenimine **16**. This is lower than the barrier for direct ring opening of the nitrene **10** itself (16 kcal/mol) (Scheme 4 and Figure 1) [13]. Thus, while both the nitrene **10** and the ketenimine **16** may undergo ring opening with rather low activation barriers, the ring opening of the ketenimine has the lowest barrier. Both of these reactions can take place with ease under conditions of FVT.

**Scheme 5:**
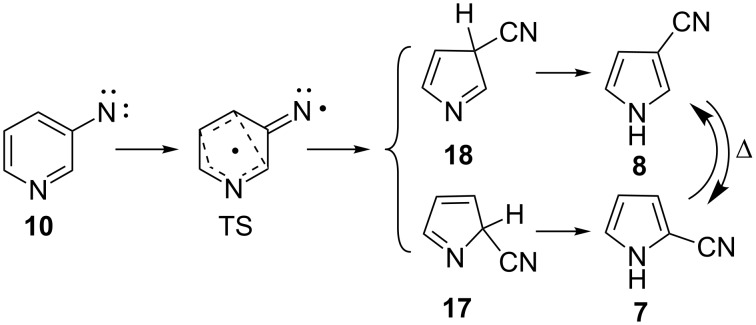
Potential direct ring contraction in 3-pyridylnitrene (**10**).

With a low barrier for ring opening, recyclization of the nitrile ylide **11** now becomes the energetically most favourable mechanism of formation of 3-cyanopyrrole (**8**) via the 3*H* tautomer **18** with a barrier of only 10 kcal/mol above the S_1_ nitrene or 16 kcal/mol above the nitrile ylide (Scheme 4 and Figure 1). This explains why 3-cyanopyrrole (**8)** is the predominant isomer, in contrast to the reaction of 2-pyridylnitrene, where 2-cyanopyrrole is formed preferentially [11]. In the analogous case of 3-quinolylnitrene, 3-cyanoindole is formed exclusively under mild FVT conditions [21]. A direct, concerted ring contraction in 3-pyridylnitrene would be possible (via **17** and **18**, Scheme 5), but such reactions have considerably higher activation barriers, ca. 30 kcal/mol in the case of phenylnitrene [22]. A transition state for the concerted ring contraction of 3-pyridylnitrene (**10**) to 3*H*-3-cyanopyrrole (**18**) was not found, but we calculate a barrier of 23 kcal/mol for the concerted ring contraction to 2-cyano-2*H*-pyrrole (**17**, Figure 1). Furthermore, a higher proportion of 2-cyanopyrrole would be expected if Scheme 5 was operating. Concerted ring contraction in the 7-membered ring ketenimine **16** is also possible [22], but this type of reaction has an even higher activation energy. The lowest-energy path for (di)azacycloheptatetraenes to undergo ring contraction is by ring opening.

**Scheme 6:**
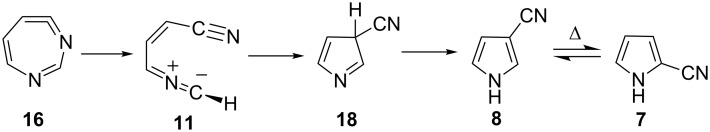
Ring contraction by ring opening to nitrile ylide **11**.

The nitrile ylide **11** is not directly observable under FVT conditions, because the barriers for its reactions are very low. The calculated barrier for cyclization to 3-cyano-3*H*-pyrrole (**18**) is 16 kcal/mol (Scheme 6 and Figure 1).

### 2- and 4-pyrimidinylcarbenes

**Scheme 7:**
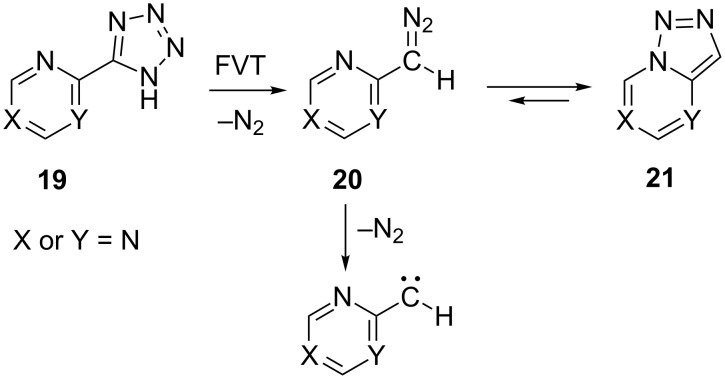
Generation of pyrimidinyldiazomethanes and pyrimidinylcarbenes.

FVT of tetrazolylpyrimidines of type **19** causes elimination of N_2_ to generate 4- and 2-diazomethylpyrimidines **20**, which can ring-close to the corresponding 1,2,3-triazolopyrimidines **21** (Scheme 7) [23]. Endothermic ring-chain valence isomerization of the type **21** → **20**, with free energies of activation of 18–22 kcal/mol in solution, has been demonstrated for 1,2,3-triazolo[1,5-*a*]pyrimidines [24] but not for 1,2,3-triazolo[1,5-*c*]pyrimidines [25–26]. However, 7-benzyl-3-ethoxycarbonyl-1,2,3-triazolo[1,5-*c*]pyrimidin-5-ol and its diazo valence tautomer, 6-(2-diazoethoxycarbonylmethylene)-2-(α-hydroxybenzyl)pyrimidin-4-(2*H*)-one, have been reported [27].

**Scheme 8:**
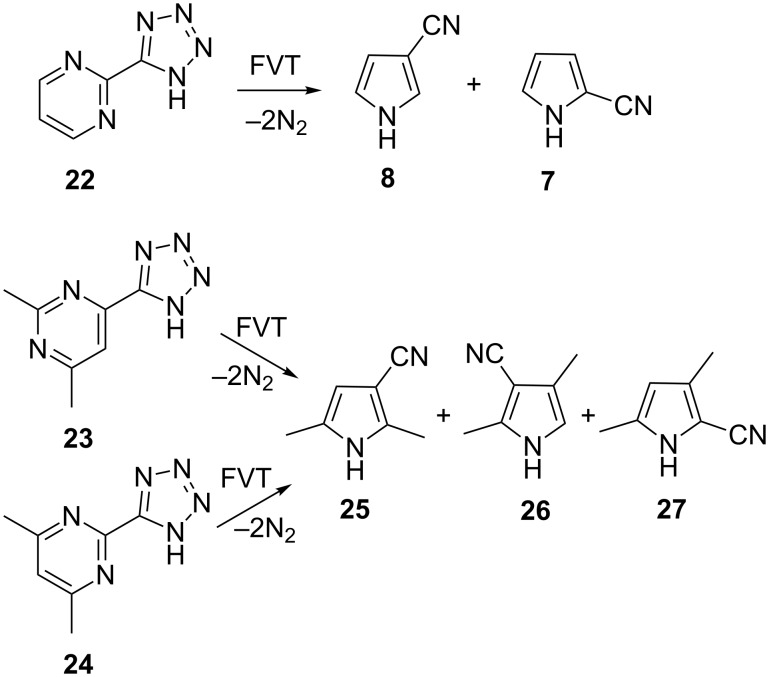
Formation of cyanopyrroles by FVT of tetrazolylpyrimidines.

We find that FVT of the 4- and 2-(5-tetrazolyl)pyrimidines **22**–**24** also affords cyanopyrroles (Scheme 8). FVT of 2-(5-tetrazolyl)pyrimidine (**22**) affords a ca. 1:1 ratio of 2- and 3-cyanopyrroles (Scheme 8). The results of FVT of tetrazoles **23** and **24** are collected in Table 1. The formation of different mixtures of the three cyanodimethylpyrroles **25**–**27** depending on the conditions can be explained in terms of chemical activation. The formation of (hetero)arylcarbenes and their rearrangement to (hetero)arylnitrenes and cyanopyrroles are strongly exothermic reactions. Consequently, when the reaction is performed in the low-pressure gas phase, the reaction products carry excess thermal (rovibrational) energy, which facilitates the sigmatropic shifts of H, CN, and CH_3_, which will cause interconversion of the cyanopyrroles [28]. In many cases, this cannot be completely avoided, even by using the mildest possible FVT temperatures, but an increase in pressure (1 hPa N_2_) will help to remove excess thermal energy and so preserve the initial reaction products. Therefore, as seen in Table 1, it can be concluded that the dimethylcyanopyrrole **25** is the primary reaction product of **23**, and **27** is the predominant product from **24**. That chemical activation is the cause is seen in the fact that FVT of the individual dimethylcyanopyrroles **25** and **27** afford mixtures very similar to those obtained from **23** and **24**, respectively, but a temperature of 800 °C is required for this, whereas 400 °C suffices in the FVT of the tetrazoles (Table 1).

**Table 1:** Dimethylcyanopyrroles from tetrazolylpyrimidines.

Starting material	Conditions	Products, rel. yields (%)		

	*T* (°C)/*p* (hPa)	**25**	**26**	**27**

**23**	400/10^−3^	63	29	7
	400/1 N_2_	98	2	<0.5
	600/10^−1^	79	19	2
**24**	400/10^−3^	34	23	43
	400/1 N_2_	21	10	69
	600/10^−1^	25	23	44*^a^*
**25**	800/10^−3^	49	32	14*^a^*
**27**	800/10^−3^	22	22	50*^a^*

*^a^*6–8% yields of other, isomeric dimethylcyanopyrroles were also formed.

**Scheme 9:**
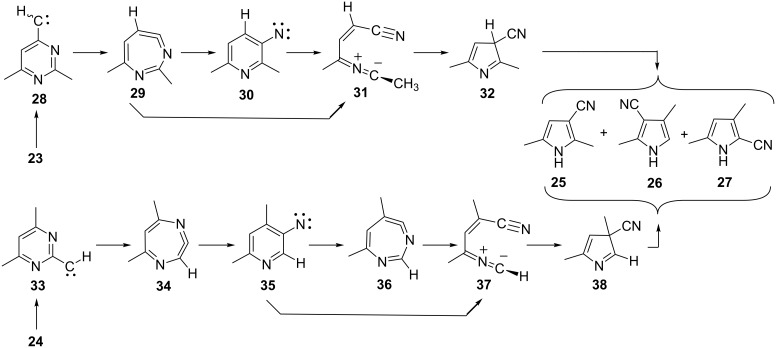
Rearrangements of pyrimidinylcarbenes to cyanopyrroles via nitrile ylides **31** and **37**.

Following the analysis of the ring contraction in 3-pyridylnitrene (**10**) by ring expansion and ring opening to a nitrile ylide (Scheme 4 and Figure 1), we can interpret the reactions in terms of ring expansion of the pyrimidinylcarbenes **28** and **33** to diazacycloheptatetraenes **29** and **34**, ring contraction to 3-pyridylnitrenes **30** and **35** and/or ring opening to nitrile ylides **31** and **37**, and ring closure to cyanopyrroles (Scheme 9).

In the case of the 4-pyrimidinylcarbene **28** a direct ring opening of the diazacycloheptatetraene **29** to the nitrile ylide **31** is possible. However, in the case of the 2-pyrimidinylcarbene **33**, the first-formed diazacycloheptatetraene **34** cannot open directly to a nitrile ylide but must first rearrange to the 3-pyridylnitrene **35**. Either the 3-pyridylnitrene **35** or the second diazacycloheptatetraene **36** may then undergo ring opening to the nitrile ylide **37**. Sigmatropic shifts of H, CN, or CH_3_ in the 3*H*-pyrroles **32** and **38** lead to the final products.

**Scheme 10:**
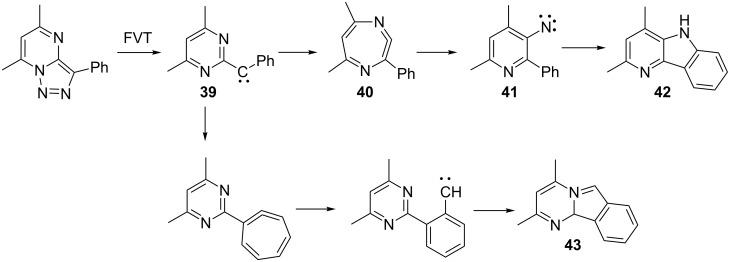
Rearrangements of phenyl(dimethylpyrimidinyl)carbene.

We have previously reported strong evidence for the ring expansion of a 2-pyrimidinylcarbene **39** to a diazacycloheptatetraene **40** and subsequent ring contraction to a 3-pyridylnitrene **41**, which undergoes cyclization to afford the pyridoindole (4-azacarbazole) **42** (Scheme 10) [29].

The alternative ring expansion/ring contraction/recyclization to the pyrimidoisoindole **43** also takes place [29]. The formation of **42** is important, as it demonstrates that 2-pyrimidinylcarbenes can rearrange to 3-pyridylnitrenes, whereas 4-pyrimidinylcarbenes do not necessarily rearrange to 3-pyridylnitrenes (see **55** below).

### 2-Phenyl-3-quinolylnitrene

**Scheme 11:**
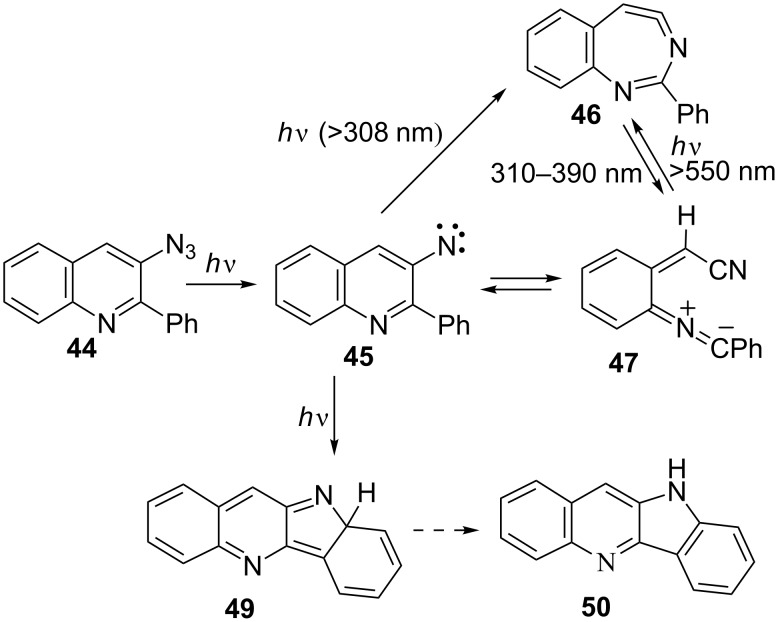
Photolysis of 3-azido-2-phenylquinoline.

**Figure 2:**
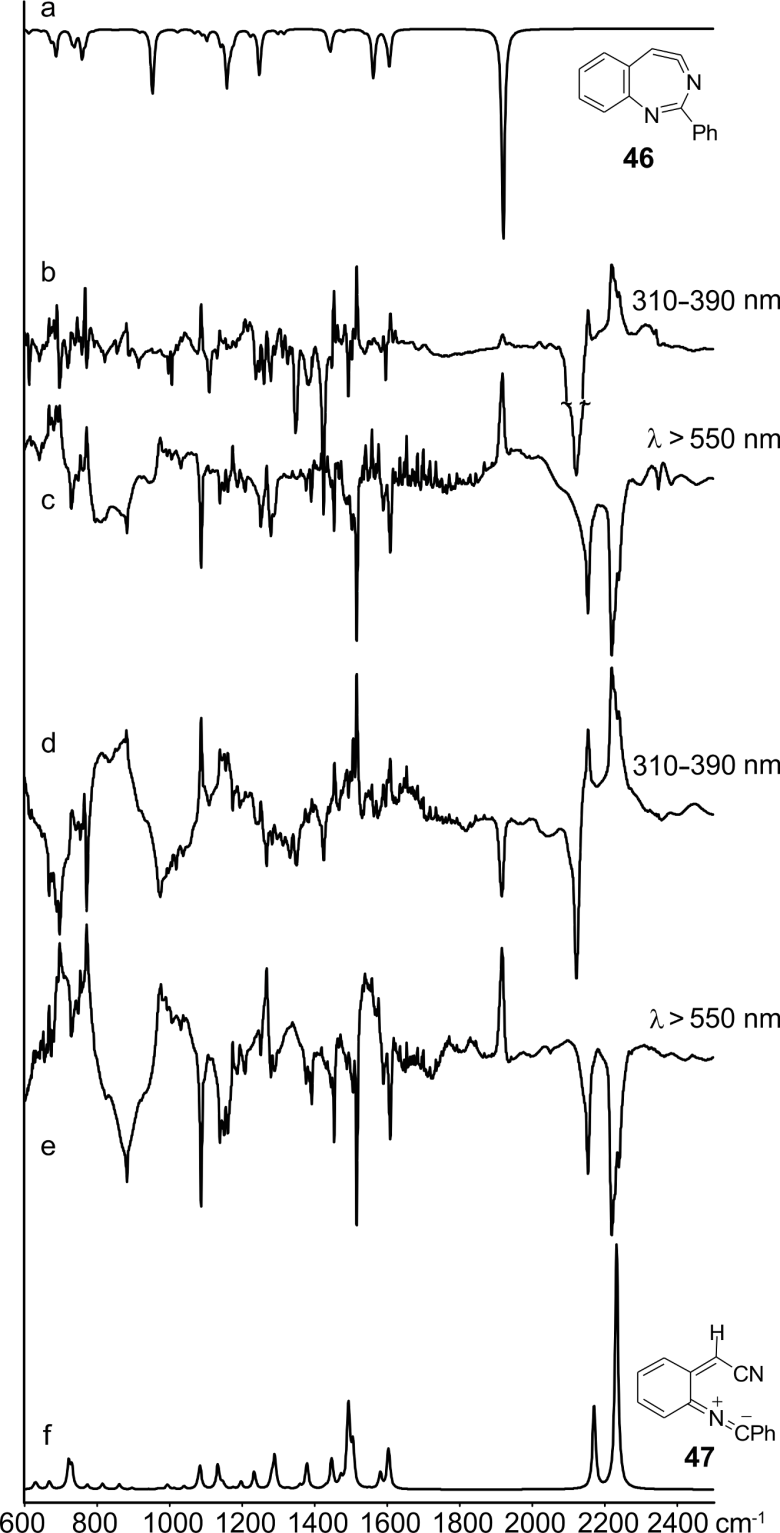
IR difference spectra from the photolysis of 3-azido-2-phenylquinoline (**44**) in Ar matrix. (a) Calculated spectrum of **46**. (b) Initial difference spectrum after 240 s; the negative peaks are due to the azide. (c)–(e) Further irradiation as indicated for 900, 400 and 600 s, respectively. (f) Calculated spectrum of **47**. Ordinate in arbitrary absorbance units. See also Figures S1–S3 in Supporting Information File 1.

**Figure 3:**
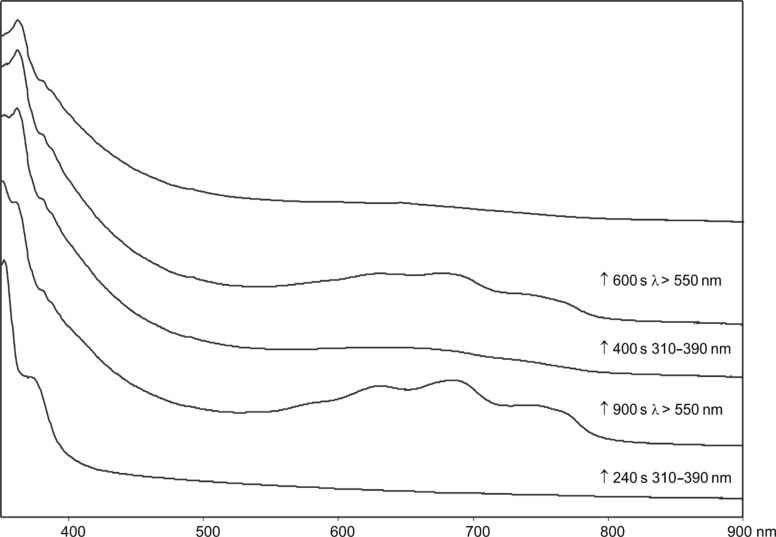
UV–vis spectra from the sequential photolysis of 3-azido-2-phenylquinoline (**44**) in Ar matrix at 310–390 nm and 550 nm. Abscissa in nanometres and ordinate in arbitrary absorbance units. These spectra correspond to the IR spectra in Figure 2. See Supporting Information File 1 for the calculated electronic transitions.

Matrix photolysis of 3-azido-2-phenylquinoline (**44**) at λ = 308 nm or 310–390 nm affords a blue nitrile ylide **47** (observed IR 2220, 2154 cm^−1^; calcd (B3LYL/6-31G*) 2233, 2171 cm^−1^; UV–vis λ_max_ 400 and 680 nm) (Scheme 11, Figure 2 and Figure 3).

It could be converted to the seven-membered ring ketenimine **46** (IR: 1920; calcd: 1921 cm^−1^) on photolysis above 550 nm. The ketenimine **46** was again converted to ylide **47** on photolysis at 310–390 nm. We assume the nitrene **45** is formed initially, but it is converted to **46** and **47** at the same wavelength. As in the examples described above, the ring opening to the ylide **47** may take place either from the nitrene or from the cyclic ketenimine, although we only observed the latter reaction directly. It was possible to cycle many times between these two intermediates (Figure 2 and Figure 3), but eventually signal intensity was lost, probably because another reaction, the cyclization to the isocarbazole **49**, took place. This reaction is analogous to the photocyclization of *o*-biphenylylnitrene to isocarbazole [30]. As shown below, the indoloquinoline **50** is indeed a major product under FVT conditions. It is possible that **49** could contribute to the observed visible spectrum, but it obviously cannot explain the IR spectrum. The same visible spectrum was obtained by photolysis of **44** embedded in a PVC film at 11 K, and the carrier of the spectrum was stable up to 90 K, as can be expected of a highly reactive nitrile ylide **47**. The calculated IR and UV–vis spectra of **45**, **46**, **47** and **49** are listed in Supporting Information File 1.

**Scheme 12:**
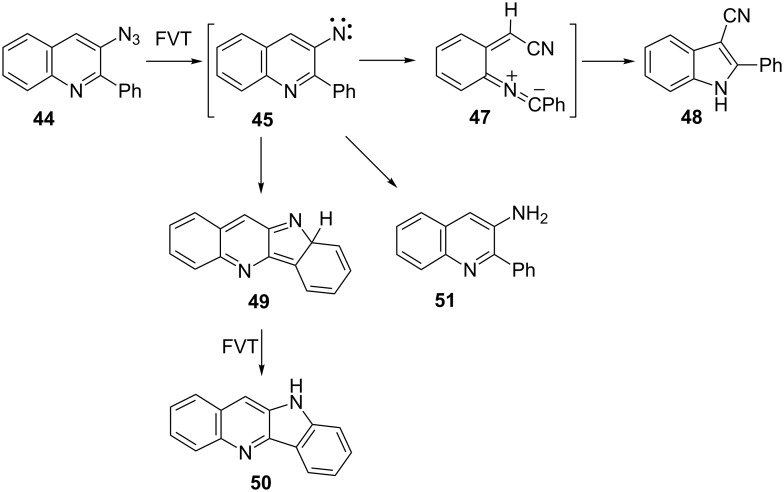
Preparative FVT of 3-azido-2-phenylquinoline.

Preparative FVT of azide **44** at 400–800 °C affords the indoloquinoline **50** in 52–60% yield (Scheme 12). Importantly, smaller amounts of amine **51** and the ring-contraction product 2-phenyl-3-cyanoindole (**48**, 21–15%) were also isolated.

Formation of amines is diagnostic for triplet nitrenes, even in low-pressure gas-phase reactions [10,22]. The 3-cyanoindole **48** is expected to be formed by cyclization of the nitrile ylide **47** in the same way that the 3-cyanoindole is obtained from the unsubstituted 3-quinolylnitrene [21]. The three products, **48**, **50** and **51** were formed even on thermolysis of **44** in solution.

### 4-Quinazolinylcarbenes

**Scheme 13:**
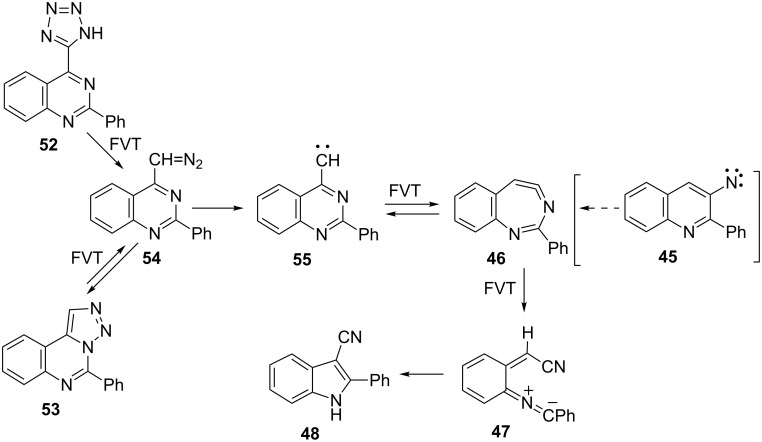
FVT of 2-phenyl-4-quinazolinylcarbene precursors.

The tetrazolylquinazoline **52** and the triazoloquinazoline **53** undergo pyrolysis via the diazomethylquinazoline **54** (Scheme 13) [23]. Both afforded the 3-cyanoindole **48** in nearly quantitative yield (up to 98%) on FVT at 400–600 °C, but no traces of either indoloquinoline **50** or the 3-aminoquinoline **51** were detectable. The same was true when the thermolysis was performed in solution. In other words, nitrene **45** was not formed (Scheme 13).

This is in agreement with the conclusion reached for 4-quinazolinylcarbene itself [21], namely that ring contraction takes place via the pathway 2-phenyl-4-quinazolinylcarbene (**55**) → cyclic ketenimine **46** → nitrile ylide **47** → nitrile **48**. The data demonstrate that carbene **55** does not convert thermally to nitrene **45**. In other words, although carbene–nitrene rearrangements are known to occur [1,19] (see, e.g., Scheme 1 and Scheme 10 above), they may be bypassed when ring opening to nitrile ylides becomes more favourable.

## Conclusion

The rearrangement of 2- and 4-pyrimidinylcarbenes **28** and **33** to mixtures of 2- and 3-cyanopyrroles on FVT implies prior isomerization to 3-pyridylnitrenes and/or diazacycloheptatetraenes and nitrile ylides. 3-Quinolylnitrenes and 4-quinazolinylcarbenes afford 3-cyanoindoles **48** on FVT. In addition, 2-phenyl-3-quinolylnitrene (**45**) cyclizes to indoloquinoline **50**, but this compound is not formed at all from the isomeric 2-phenyl-4-quinazolinylcarbene (**55**). This demonstrates that carbene **55** does not isomerize to nitrene **45**. Instead, the ring contraction to **48** takes place via the nitrile ylide **47**. Matrix photolysis of 2-phenyl-3-azidoquinoline (**44**) revealed a reversible photochemical interconversion of the benzodiazacycloheptatetraene **46** and the nitrile ylide **47**.

## Experimental

### General

The apparatus and procedures for preparative FVT [31] and for Ar matrix isolation [11,32–33] were as previously described. KBr and CsI windows were used for IR spectroscopy. FVT products were isolated in liquid nitrogen (77 K) in the preparative thermolysis, and at 22–25 K in Ar matrices for IR experiments. IR spectra of the Ar matrices were measured at 7–10 K with a resolution of 1 cm^−1^. Photolyses were done through quartz by using a 75 W low pressure Hg lamp (254 nm) or a 1000 W high pressure Hg/Xe lamp equipped with a monochromator and appropriate filters. A water filter was used to remove infrared radiation, and a 7.5% NiSO_4_ or a NiSO_4_/CuSO_4_ solution (7.5 and 2.5%, respectively) to remove visible light where required. Analytical gas chromatography used a 10% OV 17 column programmed at 120–180 °C at 2°/min with N_2_ as carrier gas at 0.6 bar. Preparative gas chromatography used a 20% Carbowax column at 200 °C with H_2_ as carrier gas at 60 mL/min.

#### Materials

3-Azidopyridine (**9**) [34–35], 2-(5-tetrazolyl)pyrimidine (**22**) [36–37], 2-phenyl-4-(5-tetrazolyl)quinazoline (**52**) [23], and 5-phenyl-1,2,3-triazolo[1,5-*a*]quinazoline (**53**) [23] were prepared according to literature procedures. 4,6-Dimethyl-2-(5-tetrazolyl)pyrimidine (**24**) and 2,4-dimethyl-6-(5-tetrazolylpyrimidine) (**23**) were prepared from the dimethylpyrimidine-carbonitriles [38–39] by adaptation of the literature method [36–37].

**4,6-Dimethyl-2-(5-tetrazolyl)pyrimidine (24):** From 3.0 g (22.5 mmol) of 4.6-dimethylpyrimidine-2-carbonitrile was obtained 2.8 g (71%); mp 209–210 °C (dec); ^1^H NMR (CDCl_3_) 12.0 (very broad), 7.37 (s, 1H), 2.50 (s, 6H); MS *m/z*: 176 (M^+^, 21), 148 (33), 134 (46), 120 (100), 119 (71), 107 (36), 105 (12), 93 (13), 92 (16 ), 80 (15), 67 (76), 66 (56), 53 (26); anal. calcd for C_7_H_8_N_6_: C, 47.72; H. 4.58; N, 47.70; found: C, 47.89; H, 4.40; N, 47.52.

**2,4-Dimethyl-6-(5-tetrazolyl)pyrimidine (23):** Prepared as the preceding entry in 41% yield; mp 208–209 °C (dec); ^1^H NMR (CDCl_3_) 7.43 (s, 1H), 2.66 (s, 3H), 2.56 (s, 3H); MS *m/z*: 176 (M^+^, 49), 148 (12), 135 (10), 134 (100), 120 (19), 119 (65), 107 (42), 93 (17), 97 (15), 78 (17), 66 (66), 52 (54); anal. calcd for C_7_H_8_N_6_: C, 47.72; H. 4.58; N, 47.70; found: C, 47.90; H, 4.80; N, 47.75.

**3-Azido-2-phenylquinoline (44):** This compound was prepared according to a standard literature procedure [40]. A sample of 2.54 g (11.5 mmol) of 3-amino-2-phenylquinoline [41] was dissolved in a mixture of 2 mL of conc. sulfuric acid (*d* = 1.84) and 13 mL water at 40 °C and then cooled to 0 °C. A solution of 900 mg (13 mmol) of NaNO_2_ in 8 mL water was added dropwise at 0 °C, causing the formation of a yellow, crystalline diazonium sulfate, which was not isolated. After 30 min at 0 °C a solution of 11 g (16.9 mmol) of NaN_3_ in 7 mL water was added. An amorphous, yellow precipitate formed immediately with simultaneous evolution of N_2_. After stirring for 3 h at rt, the yellow solid was filtered and recrystallized from petroleum ether, yielding 2.33 g (82%) of the azide, mp 93–94 °C; ^1^H NMR (DMSO-*d*_6_) 8.41 (s, 1H), 8.05–8.01 (m, 2H), 7.87–7.83 (m, 2H), 7.73 (dd, *J* = 8.2 Hz, *J* = 1.2 Hz, 1H), 7.64 (dd, *J* = 8.2 Hz, *J* = 1.2 Hz, 1H), 7.53–7.47 (m, 3H); ^13^C NMR (DMSO-*d*_6_) 151.4, 144.0, 137.3, 132.2, 129.6, 129.1, 128.9, 128.7, 127.9, 127.5, 127.4, 126.7, 124.9; IR (KBr): 3030 (w), 2100 (s), 1590 (w), 1485 (m), 1415 (s), 1340 (s), 1288 (m), 1270 (s), 1255 (m), 1245 (m), 1230 (m), 955 (m), 895 (s), 860 (m), 770 (s), 750 (s), 710 (s), 695 (s) cm^−1^; UV (CH_3_CN) λ_max_: 257, 260, 337 nm; MS *m/z*: 246 (M^+^, 5), 220 (9), 219 (26), 218 (100), 190 (8), 115 (20), 88 (9), 78 (3); anal. calcd for C_15_H_10_N_4_: C, 73.16; H, 4.09; N, 22.75; found: C, 73.10; H, 4.18; N, 22.68.

**FVT of 3-azidopyridine (9):** The azide (0.50 g) was distilled into the FVT apparatus from a sample flask held at −30 °C and thermolysed at 370–500 °C/10^−3^–10^−4^ mbar in the course of 2 h. The pyrolysate was examined by GC/MS and ^1^H NMR spectroscopy. The 2- and 3-cyanopyrroles **7** and **8** were separated by flash chromatography on silica gel 60, eluted with hexane/ethyl acetate 3:7 and identified by comparison with authentic materials reported previously [10,20]. Yields were determined by GC as previously described [10]. The following ratios of 3- to 2-cyanopyrroles at different temperatures were obtained: 370 °C: 2.29:1 (81% 3-azidopyridine remained unreacted); 400 °C: 1.63:1 (36% 3-azidopyridine remained unreacted); 500 °C: 1.48:1 (6% 3-azidopyridine remained unreacted). GC/MS retention times and molecular masses: 2-cyanopyrrole 3.60 min (*m/z* 92); 3-cyanpyrrole 5.45 min (*m/z* 92); 3-azidopyridine 2.19 min (*m/z* 120); ^1^H NMR (CDCl_3_) 2-cyanopyrrole: 9.01 (br, 1H), 6.98 (m, 1H), 6.88 (m, 1H), 6.28 (m, 1H); 3-cyanopyrrole: 8.80 (br, 1H), 7.36 (m, 1H), 6.84 (m, 1H), 6.53 (m, 1H); IR (Ar, 20 K): 2-cyanopyrrole: 2236 cm^−1^; 3-cyanopyrrole: 2234 and 2247 cm^−1^.

**FVT of 2-(5-tetrazolyl)pyrimidine (22):** A portion of **22** (300 mg) was sublimed into the pyrolysis tube at 170 °C and pyrolysed at 600 °C/10^−3^ mbar in the course of 12 h. The resulting pyrolysate (120 mg) was identified as a 1:1 mixture of 2- and 3-cyanopyrroles **7** and **8** by comparison of the ^1^H NMR, IR and mass spectra with those of authentic materials. Yields were determined by GC as above [10].

**FVT of 2,4-dimethyl-6-(5-tetrazolyl)pyrimidine (23) and 4,6-dimethyl-2-(5-tetrazolyl)pyrimidine (24):** A portion of **23** or **24** (500 mg) was sublimed at 150 °C and pyrolysed at 10^−3^ mbar in the course of 12–18 h. The pyrolyzates were extracted with diethyl ether, and the products were analysed and separated by GC on the Carbowax column. The yields of 2,6-dimethyl-3-cyanopyrrole (**25**), 2,4-dimethyl-3-cyanopyrrole (**26**) and 3,5-dimethyl-2-cyanopyrrole (**27**) at various temperatures are collected in Table 1. Yields were determined by GC, and the pyrroles were characterized as follows:

**2,6-Dimethyl-3-cyanopyrrole (25):** Retention time 63 min. ^1^H NMR (CDCl_3_) 8.4 (br, 1H), 5.95 (m, 1H), 2.38 (s, 3H), 2.20 (s, 3H); IR (KBr): 3470 (br), 2210 (s), 1600 (w), 1430 (w), 1050 (m), 780 (m); UV (EtOH) λ_max_: 245 nm; MS *m/z*: 120 (M^+^, 67), 119 (100), 106 (28), 105 (71), 78 (10), 65 (3). The spectra data were in agreement with literature values [42].

**2,4-Dimethyl-3-cyanopyrrole (26):** Retention time 57 min; ^1^H NMR (CDCl_3_) 8.60 (br, 1H), 6.38 (m, 1H), 2.38 (s 3H), 2.12 (s, 3H); IR (KBr): 3460 (br), 2210 (s), 1580 (w), 1400 (m), 1110 (m), 800 (m); MS *m/z*: 120 (M^+^, 55), 119 (100), 106 (25), 105 (60), 78 (12), 65 (3); UV (EtOH) λ_max_: 240 nm. The spectra data were in agreement with literature values [42].

**3,5-Dimethyl-2-cyanopyrrole (27):** Residence time 31 min; ^1^H NMR (CDCl_3_) 9.0 (br, 1H), 5.76 (m, 1H), 2.23 (s, 3H), 2.17 (s, 3H); IR (KBr): 3460 (br), 2210 (s), 1580 (w), 1460 (w), 1390 (w), 1300 (w), 1270 (m), 800 (m); MS *m/z*: 120 (M^+^, 73), 119 (100), 106 (48), 105 (86), 92 (8), 78 (14), 65 (10); UV (EtOH) λ_max_: 257, 234 nm. The spectra data were in agreement with literature values [42]. Anal. calcd for C_7_H_8_N_2_: C, 69.97; H, 6.71; N, 23.31; found: C, 69.61; H, 6.31; N, 23.21

**Matrix photolysis of 3-azido-2-phenyl-3-quinoline (44):** The azide was sublimed at 80 °C and deposited with Ar at 22 K to form a matrix. Principal absorptions of the azide at 11 K: 2100, 1590, 1420, 1340, 1270, 1100 cm^−1^. Irradiation of the azide **44** at 308 nm or at 310–390 nm for 30 s afforded the nitrile ylide **47** together with a smaller amount of the cyclic ketenimine **46** (2220, 2154, 1920, 1610, 1516, 1500, 1450, 1390, 1088 cm^−1^). Further photolysis for 210 s caused additional formation of the ylide **47** (IR, Ar, 10 K: 2220, 2154, 1609, 1516, 1453, 1088, 740 cm^−1^ (Figure 2); UV–vis λ_max_ ca. 350 and 585, 635, 700, 775 nm (Figure 3)). Subsequent irradiation at λ > 550 nm bleached the long-wavelength band in the visible spectrum and the IR bands at 2154 and 2220 cm^−1^ in the IR. The intensity of the band at 1918 cm^−1^ due to **46** increased substantially at the same time (Figure 2). Renewed irradiation at 310–390 nm caused diminution of the IR bands ascribed to **46** and reformation of the IR and UV–vis bands ascribed to the nitrile ylide **47**. It was possible to cycle several times between these two species by using λ > 550 nm and λ = 310–390 nm, respectively (Figure 2 and Figure 3).

**FVT of 3-azido-2-phenyl-3-quinoline (44):** (a) A sample of the azide (0.2 g, 0.8 mmol) was sublimed at 60–80 °C and pyrolysed at 500 °C/10^−4^ mbar in the course of 3 h. The products were separated by flash chromatography on silica gel, eluting with chloroform. Indolo[3,2-*b*]quinoline (**50**) was obtained in 56–65% yield in different experiments: mp 250–251 °C (lit. [43] 248 °C; lit. [44] 251–252 °C); ^1^H NMR (DMSO-*d*_6_) 11.40 (s, 1H), 8.35 (d, *J* = 8.3 Hz, 1H), 8.27 (s, 1H), 8.18 (d, *J* = 8.3 Hz, 1H), 8.09 (d, *J* = 8.3 Hz, 1H), 7.66–7.52 (m, 4H), 7.28 (dd, *J* = 7.4 Hz, *J* = 1.0 Hz, 1H); ^13^C NMR 145.6, 144.0, 143.3, 132.4, 129.6, 128.5, 127.4, 126.6, 125.9, 124.7, 121.3, 120.9, 119.2, 113.0, 111.4; IR (KBr): 3300–3000 (br), 1610 (s), 1490 (s), 1460 (m), 1400 (s), 1340 (s), 1220 (s), 1120 (m), 750 (m), 730 (s) cm^−1^; UV (EtOH) λ_max_: 347, 276 nm; MS *m/z*: 219 (M^+^ + 1, 16), 218 (M^+^, 100), 217 (16), 190 (15), 109 (M^++^, 11), 108 (10), 96 (17), 95 (10), 89 (20), 77 (10). 3-Cyano-2-phenylindole (**48**) was obtained in 18–20% yield in different experiments: mp 241–243 °C (lit. [45] 246–248 °C, lit. [46] 240 °C); ^1^H NMR (CDCl_3_) 8.91 (br s, 1H), 7.89–7.96 (m, 2H), 7.76 (dd, *J* = 7.2 Hz, *J* = 0.6 Hz, 1H), 7.56–7.42 (m, 4H), 7.35–7.27 (m, 2H); ^13^C NMR (CDCl_3_) 144.7, 135.5, 129.9, 129.4, 129.2, 128.3, 126.9, 123.8, 122.0, 118.3, 117.0, 112.6, 81.4; IR (KBr): 3240–3180 (br s), 2220 (s), 1490 (m), 1450 (s), 1420 (m), 1370 (w), 1320 (br w), 1240 (m), 770 (m), 730 (s), 710 (w), 680 (m); MS *m/z*: 219 (M^+^ + 1, 17), 218 (M^+^, 100), 190 (9), 115 (5), 109 (8), 96 (6). (b) In analogous experiments at 450–700 °C the products of FVT were condensed on a KBr window at 77 K for IR spectroscopy. In each case the product was a mixture of 3-cyano-2-phenylindole and indolo[3,2-*b*]quinoline according to IR spectroscopy of the crude and TLC of the isolated material.

**Thermolysis of 3-azido-2-phenylquinoline (44) in solution**: A solution of 200 mg (0.81 mmol) of the azide in xylene was heated under reflux for 3 days. After distillation of the solvent, the residue was chromatographed on silica, eluting with petroleum ether/chloroform to yield 3-cyano-2-phenylindole (**48**, 3 mg; 2%), 3-amino-2-phenylquinoline (**51**, 80 mg; 45%), and indolo[3,2-*b*]quinoline (52 mg; 29%).

**FVT of 2-phenyl-4-(5-tetrazolyl)quinazoline (52):** (a) A sample of 300 mg (1.10 mmol) was sublimed at 150 °C and pyrolysed at 600 °C/10^−3^–10^−4^ mbar in the course of 12 h. Chromatography yielded 178 mg (75%) of 3-cyano-2-phenylindole (**48**). (b) In similar experiments with FVT at 600–800 °C the product was isolated on a KBr window at 77 K for IR spectroscopy. In each case 3-cyano-2-phenylindole (**48**) was the exclusive product.

**Thermolysis of 2-phenyl-4-(5-tetrazolyl)quinazoline (52) in solution:** (a) A solution of 250 mg (0.91 mmol) in 50 mL of xylene was heated under reflux for 6 d. Chromatography after distillation of the solvent yielded 166 mg (74%) 2-phenyl-1,2,3-triazolo[1,5-*c*]quinazoline **53**, mp 192–193 °C (dec) (lit. [23] 192–193 °C (dec)). (b) A solution of 50 mg (0.18 mmol) in 10 mL of diphenylmethane was heated at 180 °C for 1 h. After distilling the solvent vacuum and chromatography of the residue, 6 mg (15%) of 3-cyano-2-phenylindole (**48**) was obtained.

**FVT of 2-phenyl-1,2,3-triazolo[1,5-*c*]quinazoline (53):** (a) A sample of 50 mg (0.20 mmol) was sublimed at a temperature increasing gradually to 190 °C and pyrolysed at 660 °C/10^−3^ mbar. The product (43 mg; 98%) consisted exclusively of 3-cyano-2-phenylindole (**48**). (b) In similar experiments with FVT at 300–600 °C the product was isolated on a KBr window at 77 K for IR spectroscopy. No reaction was observable below 400 °C. The 4-diazomethyl-2-phenylquinazoline (**54**) was detectable by absorption at 2095 cm^−1^ in the 400 °C experiment. In each case, at FVT temperatures of 400–600 °C, 3-cyano-2-phenylindole (**48**) was the only other identified product, characterized by its absorption at 2220 cm^−1^.

**Thermolysis of 2-phenyl-1,2,3-triazolo[1,5-*c*]quinazoline (53) in solution:** A solution of 20 mg (0.08 mmol) in 5 mL of diphenylmethane was heated at 180 °C for 1 h. Chromatography of the resulting mixture yielded 2 mg (10%) of 3-cyano-2-phenylindole (**48**).

## Supporting Information

File 1 Computational details, calculated and experimental IR spectra, and calculated electronic transitions.
