# Supplementary material for: 3-Pyridylnitrene, 2- and 4-pyrimidinylcarbenes, 3-quinolylnitrenes, and 4-quinazolinylcarbenes. Interconversion, ring expansion to diazacycloheptatetraenes, ring opening to nitrile ylides, and ring contraction to cyanopyrroles and cyanoindoles
Source: Beilstein J Org Chem. 2013 Apr 17;9:743–53. doi: 10.3762/bjoc.9.84 (PMC3678507; doi:10.3762/bjoc.9.84)
Supplement: File 1 — Computational details, calculated and experimental IR spectra, and calculated electronic transitions. [file Beilstein_J_Org_Chem-09-743-s001.pdf]

Supporting Information

for

3-Pyridylnitrene, 2- and 4-pyrimidinylcarbenes, 3-quinolylnitrenes, and 4-quinazolinylcarbenes. Interconversion, ring expansion to diazacycloheptatetraenes, ring opening to nitrile ylides, and ring contraction to cyanopyrroles and cyanoindoles

Curt Wentrup*[1], Nguyen Mong Lan[2], Adelheid Lukosch[3], Pawel Bednarek[1] and David Kvaskoff[1]

Address: [1]School of Chemistry and Molecular Biosciences, The University of Queensland, Brisbane, Qld 4072, Australia, [2]Institut de Chimie Organique, Université de Lausanne, CH-1009 Lausanne, Switzerland and [3]Fachbereich Chemie der Universität Marburg, D-3550 Marburg, Germany

Email: Curt Wentrup* - wentrup@uq.edu.au

* Corresponding author

Computational details, calculated and experimental IR spectra, and calculated electronic transitions

Contents:

Computational method S1

Figure S1: Calculated IR spectrum of nitrile ylide **47** S2

Figure S2: Calculated IR spectrum of diazacycloheptatetraene **46** S3

Figure S3: IR-difference spectra from the Ar matrix photolyses of 3-azido-2-phenylquinoline S4

Cartesian coordinates, energies and frequencies, and electronic transitions S5

References S25

Computational method

Calculations of IR spectra were carried out at the B3LYP/6-31G* level using the Gaussian 03 program package [1]. Calculated wavenumbers were scaled by a factor of 0.9613 [2]. UV-vis electronic transitions were calculated at the TD-B3LYP/6-31G* level. Energies of open-shell singlet nitrenes (S_1) were estimated using the Cramer–Ziegler method [3], which has been shown to produce reasonable values for arylnitrenes [4].

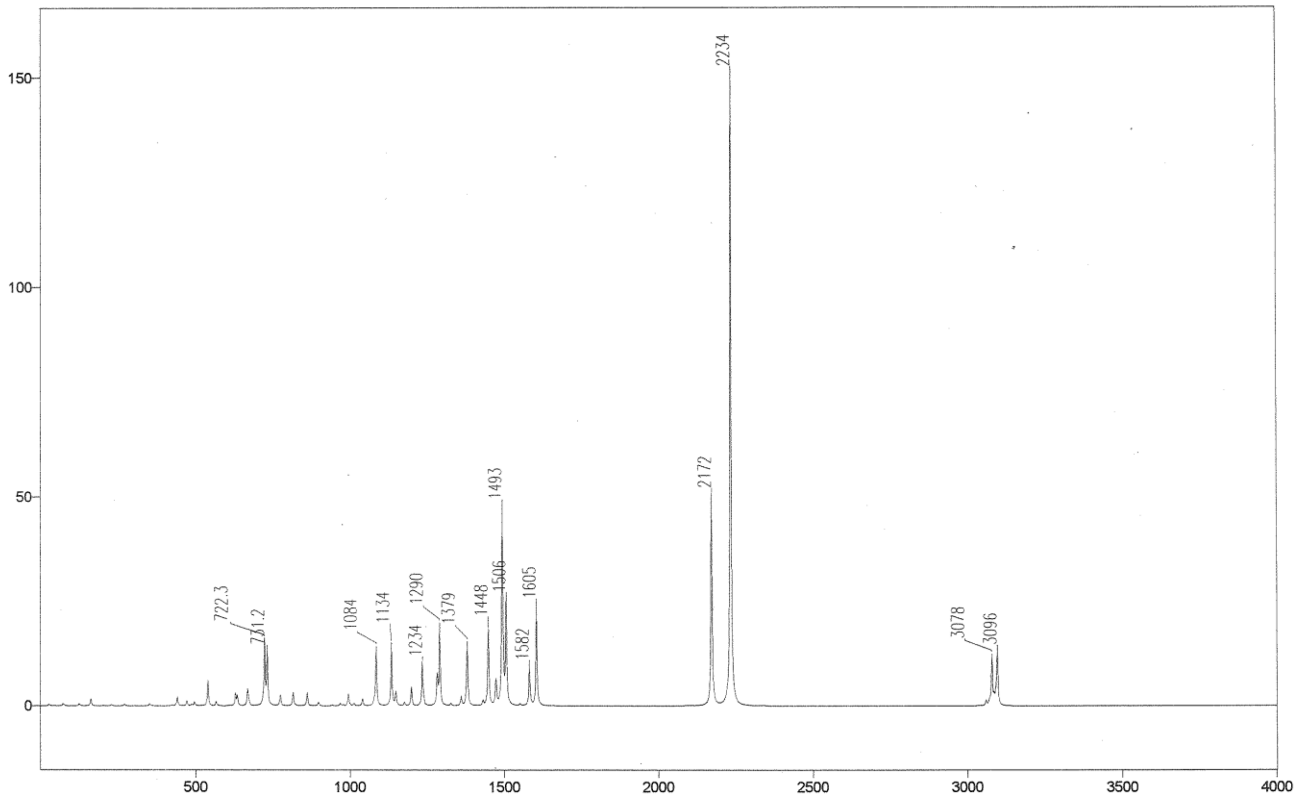

Figure S1: Calculated IR spectrum of nitrile ylide **47** at the B3LYP/6-31G* level (abscissa wavenumbers scaled by 0.9613 [2]).

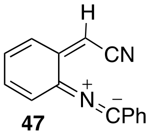

47

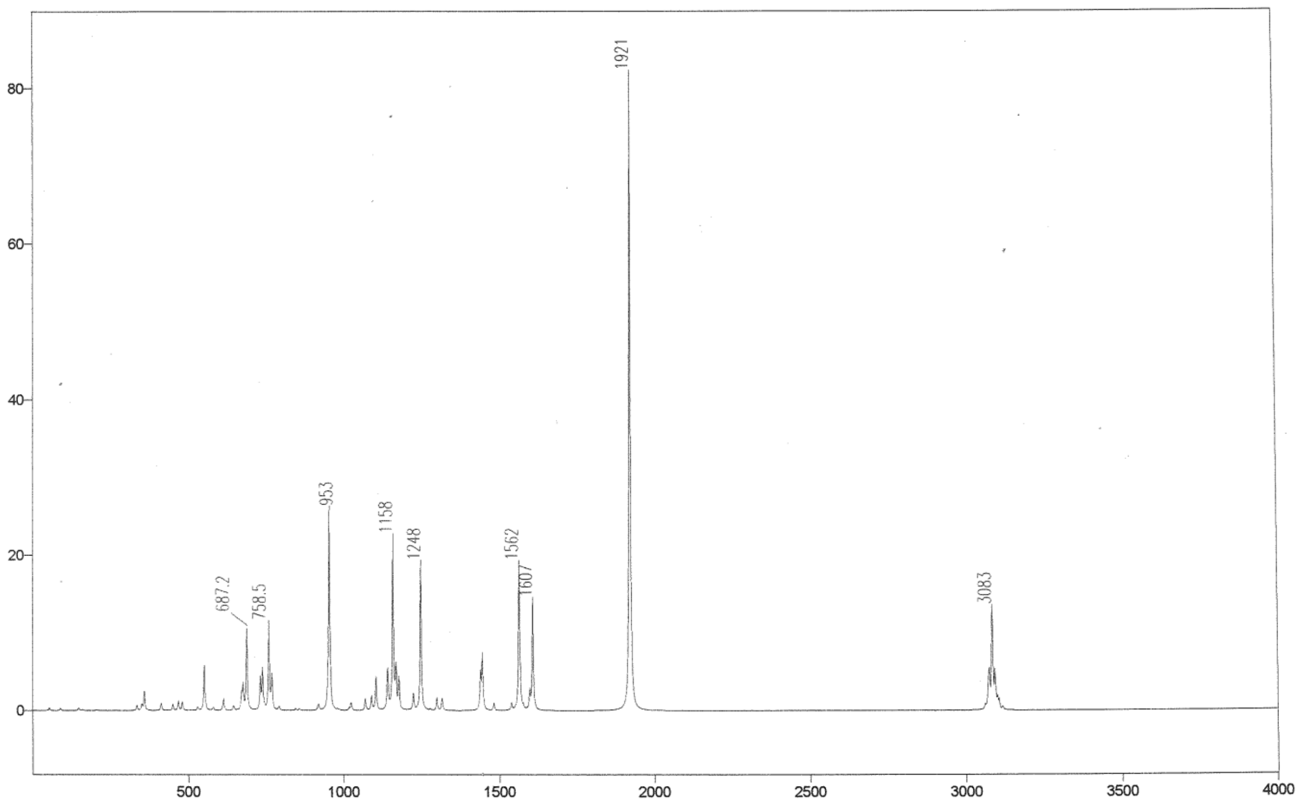

Figure S2: Calculated IR spectrum of diazacycloheptatetraene **46** at the B3LYP/6-31G* level (abscissa in wavenumbers scaled by 0.9613 [2]).

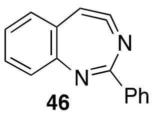

46

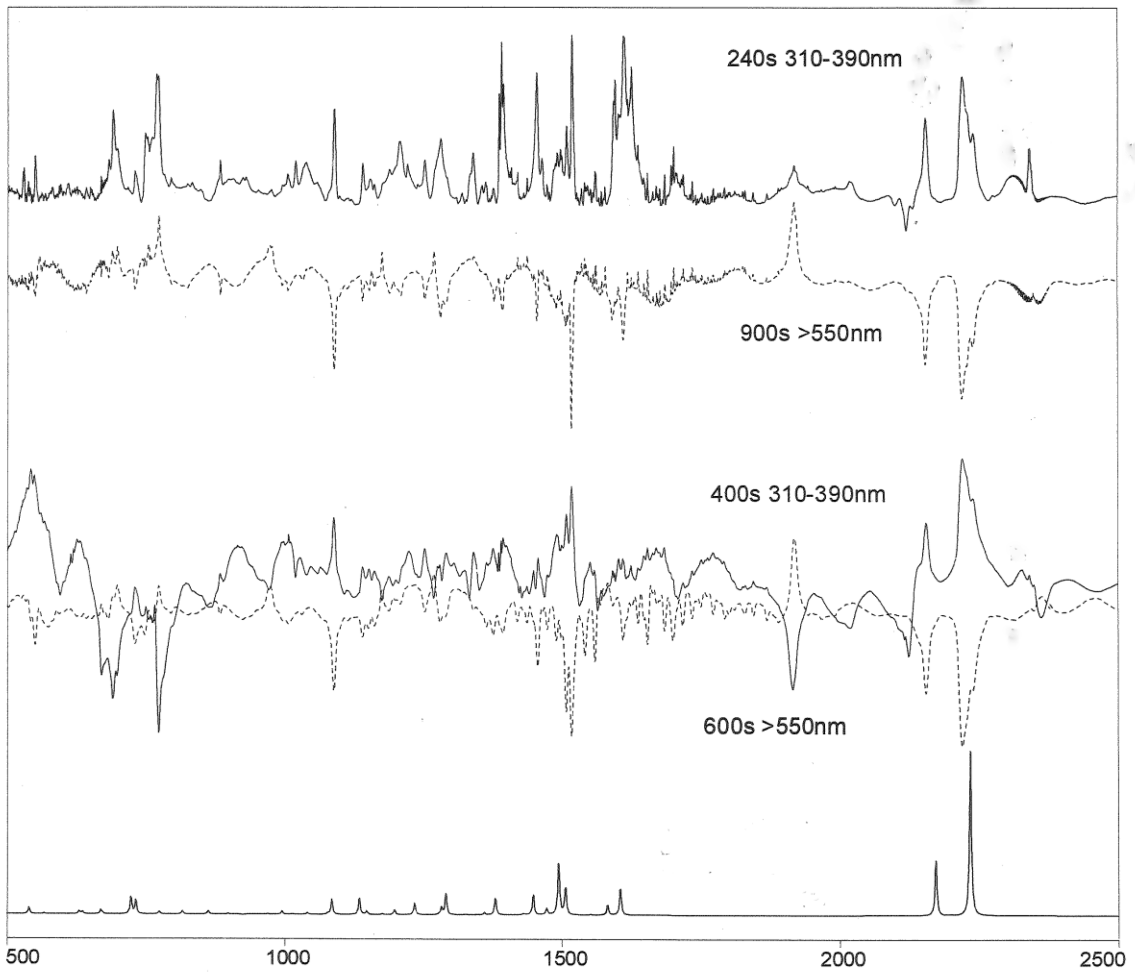

Figure S3: IR-difference spectra from the initial Ar matrix IR spectrum from photolysis of 3-azido-2-phenylquinoline (**44**) at 310–390 nm for 240 s (top; the strong azide peak in the negative spectrum has been subtracted almost completely; a positive peak at 2340 cm^{-1} is due to CO_2). Nitrile ylide **47** (2220, 2154 cm^{-1}) and cyclic ketenimine **46** (1920 cm^{-1}) have formed. Subsequent spectra are at λ > 550 nm (**46** increases; **47** decreases), 310–390 nm (**46** disappears, **47** reappears), and >550 nm (the reverse is observed) for the times indicated. Bottom: the clculated spectrum of the nitrile ylide **47** at the B3LYP/6-31G* level (wavenumbers scaled by 0.9613 [2]).

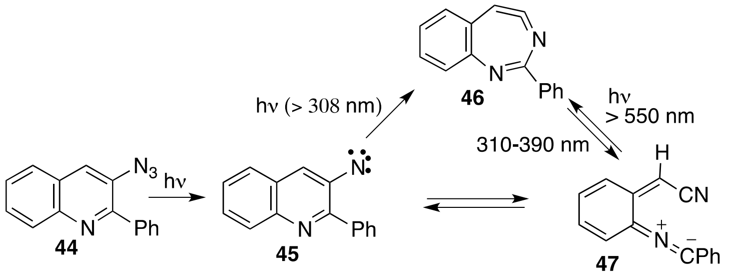

Cartesian coordinates, energies and frequencies

Method: B3LYP/6-31G*
Triplet 3-pyridylnitrene, 10 T0

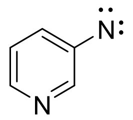


```
UB3LYP/6-31G*; Gaussian 03, Revision B.05
Point group: Cs
State=3-A", S2=2.049998
HF= -302.3423741 Hartree
Zero-point correction= 0.079545 (Hartree/Particle)
Sum of electronic and zero-point Energies= -302.292633 Hartree
```

N	-1.23223600	-1.04932800	0.00000000
N	-0.04620100	2.38012100	0.00000000
C	-1.21155300	0.27345000	0.00000000
C	0.00000000	1.05688100	0.00000000
C	1.23364700	0.32872300	0.00000000
C	1.19063000	-1.05201300	0.00000000
C	-0.05417500	-1.70070400	0.00000000
H	2.16982200	0.87753800	0.00000000
H	-2.16711000	0.79254800	0.00000000
H	2.10565100	-1.63689500	0.00000000
H	-0.11059800	-2.78676800	0.00000000

Vibrational frequencies (scaled by 0.9613):

ModeNr.	Symmetry	Wavenumber	Abs.Int.	Rel.Int.
1	A"	195.1	3.0	9
2	A'	358.1	10.3	32
3	A"	378.8	1.1	3
4	A"	459.3	3.5	11
5	A'	520.7	2.3	7
6	A'	598.2	2.6	8
7	A"	662.9	21.1	66
8	A"	767.5	25.0	78
9	A'	808.5	0.3	1
10	A"	868.0	1.0	3
11	A"	901.7	2.6	8
12	A'	945.0	18.9	59
13	A"	946.8	0.1	0
14	A'	1014.3	0.8	3
15	A'	1083.9	4.6	14
16	A'	1165.5	3.4	11
17	A'	1209.5	12.6	39
18	A'	1268.0	17.9	56
19	A'	1296.6	1.4	4
20	A'	1355.1	5.8	18
21	A'	1418.1	0.3	1
22	A'	1498.0	14.8	46
23	A'	1519.1	32.0	100
24	A'	3067.2	10.4	32
25	A'	3072.8	8.3	26

26	A'	3085.1	12.0	38
27	A'	3101.3	5.4	17

Open-shell singlet 3-pyridylnitrene, 10 S1

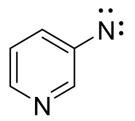

UB3LYP/6-31G*; Gaussian 03, Revision B.05
Point group: Cs
State=1-A", S2=1.03541 (50:50 singlet/triplet mixture)
HF= -302.3298861 Hartree
Zero-point correction= 0.079273 (Hartree/Particle)
Sum of electronic and zero-point Energies= -302.279443 Hartree
Ziegler-Cramer corrected energy, 2*E(S1)-E(T0)= -302.266253

N	-0.05111300	2.36938500	0.00000000
N	-1.23308500	-1.04843300	0.00000000
C	-1.21938000	0.26943000	0.00000000
C	0.00000000	1.06738000	0.00000000
C	1.24123200	0.32881300	0.00000000
C	1.19460500	-1.04724600	0.00000000
C	-0.05207800	-1.70074400	0.00000000
H	2.17611100	0.87896000	0.00000000
H	-2.17452900	0.78855300	0.00000000
H	2.10895800	-1.63351200	0.00000000
H	-0.10743700	-2.78646000	0.00000000

Vibrational frequencies (scaled by 0.9613):

ModeNr.	Symmetry	Wavenumber	Abs.Int.	Rel.Int.
1	A"	180.3	3.3	12
2	A'	362.5	10.5	39
3	A"	373.6	1.1	4
4	A"	449.5	4.1	15
5	A'	516.9	4.0	15
6	A'	592.1	2.4	9
7	A"	651.0	21.9	81
8	A"	760.4	22.2	82
9	A'	792.7	0.4	2
10	A"	848.7	2.2	8
11	A"	882.3	4.0	15
12	A'	933.9	19.1	71
13	A"	943.4	0.0	0
14	A'	1006.3	0.7	3
15	A'	1078.9	3.4	13
16	A'	1156.9	2.0	7
17	A'	1210.5	10.2	38
18	A'	1283.7	2.8	10
19	A'	1322.8	11.8	44
20	A'	1353.6	12.1	45
21	A'	1408.6	0.3	1
22	A'	1487.5	13.8	51
23	A'	1516.2	27.3	101
24	A'	3070.3	6.5	24

25	A'	3076.7	8.7	32
26	A'	3085.7	14.0	52
27	A'	3105.1	3.6	13

Closed-shell singlet 3-pyridylnitrene, 10 S2

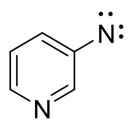

RB3LYP/6-31G*; Gaussian 03, Revision B.05
Point group: Cs
State= 1-A
HF= -302.2891322 Hartree
Zero-point correction= 0.080332 (Hartree/Particle)
Sum of electronic and zero-point Energies= -302.237761 Hartree

N	-1.24657800	-1.05987500	0.00000000
N	-0.05062000	2.38750700	0.00000000
C	-1.20440900	0.26775400	0.00000000
C	0.00000000	1.06821500	0.00000000
C	1.23443100	0.32218300	0.00000000
C	1.20244900	-1.06088700	0.00000000
C	-0.05396700	-1.68839100	0.00000000
H	2.16162700	0.88897900	0.00000000
H	-2.15157300	0.80620000	0.00000000
H	2.10519400	-1.66499600	0.00000000
H	-0.10587900	-2.77684000	0.00000000

Vibrational frequencies (scaled by 0.9613):

ModeNr.	Symmetry	Wavenumber	Abs.Int.	Rel.Int.
1	A"	144.3	10.0	5
2	A"	329.6	0.3	0
3	A'	400.4	12.9	7
4	A"	442.6	7.9	4
5	A'	536.7	2.1	1
6	A'	584.9	1.0	1
7	A"	658.0	26.4	15
8	A'	799.2	3.6	2
9	A"	800.4	16.2	9
10	A"	944.9	0.0	0
11	A'	981.0	11.9	7
12	A"	991.9	0.3	0
13	A'	1015.2	1.5	1
14	A"	1017.9	0.4	0
15	A'	1080.2	8.0	4
16	A'	1182.3	25.2	14
17	A'	1237.0	10.0	6
18	A'	1287.1	73.3	40
19	A'	1315.7	60.2	33
20	A'	1365.5	45.2	25
21	A'	1429.0	19.9	11
22	A'	1511.0	9.8	5
23	A'	1546.0	182.2	100
24	A'	3053.4	13.0	7

25	A'	3066.4	8.0	4
26	A'	3079.4	9.7	5
27	A'	3097.2	5.6	3

Azirene 14

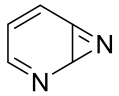

RB3LYP/6-31G*; Gaussian 03, Revision B.05
Point group: C1
State= 1-A
HF= -302.3117919 Hartree
Zero-point correction= 0.079838 (Hartree/Particle)
Sum of electronic and zero-point Energies= -302.260434 Hartree

N	0.12565800	1.46631600	0.12561300
N	1.58756500	-0.46762000	-0.66752100
C	-1.51046800	-0.36195700	-0.08180600
C	-1.05114700	1.00906000	-0.22435600
C	-0.64666500	-1.37809700	0.22537400
C	0.68828200	-0.89341700	0.10724900
C	1.09980500	0.52168700	0.42001400
H	-2.58136600	-0.54249200	-0.12002000
H	-1.80051600	1.75762600	-0.48048500
H	-0.93246900	-2.37242700	0.54925100
H	1.84294100	0.78276700	1.16576100

Vibrational frequencies (scaled by 0.9613):

ModeNr.	Symmetry	Wavenumber	Abs.Int.	Rel.Int.
1	A	243.6	18.4	40
2	A	348.7	1.2	3
3	A	362.9	5.3	12
4	A	548.3	7.7	17
5	A	580.6	12.9	28
6	A	590.2	1.6	3
7	A	627.0	18.6	40
8	A	702.1	46.1	100
9	A	846.5	5.7	12
10	A	860.0	23.6	51
11	A	894.4	0.6	1
12	A	927.3	5.7	12
13	A	941.4	15.5	34
14	A	962.8	9.2	20
15	A	997.4	8.4	18
16	A	1063.0	14.3	31
17	A	1124.2	6.6	14
18	A	1283.1	0.3	1
19	A	1339.2	4.2	9
20	A	1369.4	7.7	17
21	A	1448.3	10.5	23
22	A	1545.6	8.2	18
23	A	1762.6	29.9	65
24	A	3042.5	24.1	52

25	A	3079.3	6.1	13
26	A	3089.6	17.9	39
27	A	3108.8	2.3	5

Cyclic ketenimine 16

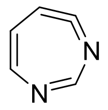

RB3LYP/6-31G*; Gaussian 03, Revision B.05
Point group: C1
State= 1-A
HF= -302.3274834 Hartree
Zero-point correction= 0.079895 (Hartree/Particle)
Sum of electronic and zero-point Energies= -302.276122 Hartree

N	0.69430000	1.24591100	0.26271000
N	1.19934600	-0.96944300	-0.50792900
C	-1.15809900	-1.04880600	0.34352400
C	-1.54470000	0.28028800	-0.11143500
C	-0.61195800	1.27806300	-0.20339600
C	1.49501000	0.25445300	0.22659700
C	0.07475100	-1.31239200	-0.09071100
H	-1.76030700	-1.70423900	0.95991400
H	-2.57503700	0.50013000	-0.38734100
H	-0.91306500	2.25790500	-0.56574100
H	2.46285000	0.30129300	0.72222900

Vibrational frequencies (scaled by 0.9613):

ModeNr.	Symmetry	Wavenumber	Abs.Int.	Rel.Int.
1	A	286.7	7.3	4
2	A	320.7	2.8	2
3	A	385.9	15.8	9
4	A	441.9	4.8	3
5	A	518.4	12.0	7
6	A	578.9	22.4	13
7	A	667.7	7.8	4
8	A	701.7	29.0	16
9	A	770.8	34.7	20
10	A	810.5	2.0	1
11	A	867.7	94.4	53
12	A	911.0	4.4	2
13	A	921.6	1.1	1
14	A	943.4	23.6	13
15	A	1023.9	5.5	3
16	A	1096.4	26.6	15
17	A	1198.0	5.8	3
18	A	1287.8	5.5	3
19	A	1315.4	20.0	11
20	A	1387.1	1.4	1
21	A	1509.9	13.4	8
22	A	1583.2	18.3	10
23	A	1881.6	177.3	100

24	A	3042.8	11.3	6
25	A	3070.8	35.1	20
26	A	3073.9	9.9	6
27	A	3115.2	1.8	1

(sZ,Z)-ylide 11

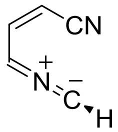

RB3LYP/6-31G*; Gaussian 03, Revision B.05
Point group: C1
State= 1-A
HF= -302.3219011 Hartree
Zero-point correction= 0.076702 (Hartree/Particle)
Sum of electronic and zero-point Energies= -302.276502 Hartree

N	-1.82572900	-1.52727800	0.07692500
N	1.47983100	-0.34027800	0.02794400
C	-1.54849000	-0.39688900	-0.00064000
C	-1.26194000	0.99286200	-0.07272400
C	-0.02274700	1.55352100	-0.00748600
C	1.27054800	0.94231800	0.09087700
C	1.67935200	-1.50215200	-0.22348500
H	-2.12661400	1.64354100	-0.16267900
H	0.01374400	2.64003200	-0.02689200
H	2.14742600	1.56590500	0.23362800
H	1.68638800	-2.31454900	0.50260800

Vibrational frequencies (scaled by 0.9613):

ModeNr.	Symmetry	Wavenumber	Abs.Int.	Rel.Int.
1	A	65.0	6.6	1
2	A	111.7	2.3	0
3	A	174.9	2.1	0
4	A	332.5	1.9	0
5	A	349.7	3.3	0
6	A	446.8	52.2	8
7	A	505.4	10.4	2
8	A	530.1	10.6	2
9	A	620.8	19.6	3
10	A	712.0	27.5	4
11	A	760.6	663.5	100
12	A	774.3	5.8	1
13	A	856.0	27.9	4
14	A	906.1	2.7	0
15	A	937.0	0.8	0
16	A	978.7	9.8	1
17	A	1177.6	4.1	1
18	A	1240.8	6.7	1
19	A	1380.4	5.7	1
20	A	1442.9	21.6	3
21	A	1576.5	73.0	11
22	A	1964.7	342.8	52

23	A	2228.4	33.2	5
24	A	3059.4	3.2	0
25	A	3064.1	7.7	1
26	A	3081.0	1.3	0
27	A	3088.0	5.0	1

2*H*-2-cyanopyrrole (17)

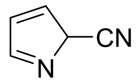

RB3LYP/6-31G*; Gaussian 03, Revision B.05
Point group: C1
State= 1-A
HF= -302.3771838 Hartree
Zero-point correction= 0.080448 (Hartree/Particle)
Sum of electronic and zero-point Energies= -302.326242 Hartree

N	0.46718100	-1.21533000	0.21982200
N	-2.70131800	0.00739100	-0.48738000
C	-1.64384000	-0.02127900	-0.01131300
C	-0.30610900	-0.01578000	0.59731100
C	0.53126300	1.18225500	0.19401900
C	1.67620000	0.70075600	-0.31657800
C	1.57225700	-0.76685500	-0.27020100
H	-0.43282300	-0.04075900	1.69120100
H	0.22514600	2.21269400	0.32413800
H	2.52317300	1.26308400	-0.69163500
H	2.34483900	-1.45402900	-0.61022300

Vibrational frequencies (scaled by 0.9613):

ModeNr.	Symmetry	Wavenumber	Abs.Int.	Rel.Int.
1	A	121.5	1.6	5
2	A	171.7	3.8	13
3	A	367.3	7.9	26
4	A	470.2	0.9	3
5	A	515.9	0.4	1
6	A	588.1	6.2	21
7	A	702.2	22.7	76
8	A	802.4	9.9	33
9	A	818.2	8.8	29
10	A	855.8	23.7	79
11	A	908.4	5.1	17
12	A	942.7	2.3	8
13	A	959.5	1.8	6
14	A	980.0	30.0	100
15	A	1007.9	9.1	30
16	A	1088.2	5.7	19
17	A	1165.1	0.4	1
18	A	1230.1	6.1	20
19	A	1275.1	0.8	3
20	A	1325.4	16.4	55
21	A	1510.9	18.0	60
22	A	1609.6	8.8	29
23	A	2282.9	5.8	19

24	A	2913.5	0.6	2
25	A	3073.5	19.1	64
26	A	3118.5	2.6	9
27	A	3141.4	2.3	8

3*H*-3-cyanopyrrole (18)

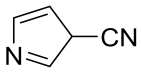

RB3LYP/6-31G*; Gaussian 03, Revision B.05
Point group: C1
State= 1-A
HF= -302.3739672 Hartree
Zero-point correction= 0.080129 (Hartree/Particle)
Sum of electronic and zero-point Energies= -302.323424 Hartree

N	1.67682300	-0.78867100	-0.30887900
N	-2.72956900	0.01199300	-0.47342100
C	0.54258500	-1.15198300	0.16826200
C	-1.66971500	0.01987000	-0.00001900
C	-0.33276000	0.02420100	0.59685500
C	0.55722500	1.17779100	0.18539800
C	1.68597900	0.64019000	-0.30357500
H	0.25130200	-2.19281200	0.27741600
H	-0.44513300	-0.00528900	1.69375700
H	0.29780100	2.22165800	0.29767600
H	2.56536300	1.15277800	-0.67427100

Vibrational frequencies (scaled by 0.9613):

ModeNr.	Symmetry	Wavenumber	Abs.Int.	Rel.Int.
1	A	118.6	5.1	17
2	A	162.9	6.8	23
3	A	369.9	1.8	6
4	A	459.3	4.1	14
5	A	509.0	2.6	9
6	A	584.0	8.8	29
7	A	706.2	18.9	63
8	A	750.0	2.6	9
9	A	798.3	3.9	13
10	A	850.0	10.9	36
11	A	889.4	29.7	99
12	A	917.3	0.0	0
13	A	962.0	7.8	26
14	A	990.0	9.2	31
15	A	1022.6	1.5	5
16	A	1102.3	3.3	11
17	A	1152.9	14.4	48
18	A	1223.0	1.5	5
19	A	1250.6	1.8	6
20	A	1313.9	6.2	21
21	A	1535.0	5.6	19
22	A	1592.8	29.8	99
23	A	2276.8	14.7	49
24	A	2899.2	1.7	6
25	A	3097.1	3.5	12

26	A	3126.0	5.3	18
27	A	3152.3	3.6	12

TS Nitrene 10 – azirene 14

```
RB3LYP/6-31G*; Gaussian 03, Revision B.05
Point group: C1
State= 1-A
HF= -302.2933825 Hartree
Zero-point correction= 0.078495 (Hartree/Particle)
Sum of electronic and zero-point Energies= -302.243293 Hartree

 N                  -0.80015800   -1.34788200    0.03202500
 N                   2.23592100    0.06346900    0.32091900
 C                  -1.20600400    1.04593200    0.03153000
 C                  -1.62108700   -0.31200400    0.16490200
 C                   0.11230000    1.34949200   -0.14577600
 C                   1.06554000    0.24543400   -0.12702700
 C                   0.47784000   -1.11313600   -0.26519900
 H                  -1.96185700    1.82610900    0.03194300
 H                  -2.68010100   -0.54422400    0.25659400
 H                   0.45984200    2.36079200   -0.33278500
 H                   1.16023200   -1.94609600   -0.37693600
```

Imaginary frequency= -84.7185 **cm-1**

TS azirene 14 – cyclic ketenimine 16

```
B3LYP/6-31G*; Gaussian 03, Revision B.05
Point group: C1
State= 1-A
HF= -302.3104574 Hartree
Zero-point correction= 0.078607 (Hartree/Particle)
Sum of electronic and zero-point Energies= -302.260144 Hartree

 N                   0.17313600    1.43610800    0.18220800
 N                   1.54559000   -0.49214500   -0.65659300
 C                  -1.51352900   -0.29750500   -0.10746100
 C                  -1.02063000    1.02907200   -0.24094600
 C                  -0.68459000   -1.35128900    0.27014900
 C                   0.63783100   -0.99300000    0.03936300
 C                   1.14668100    0.54172800    0.38846100
 H                  -2.58739200   -0.45137600   -0.16839000
 H                  -1.73066000    1.81589400   -0.48624900
 H                  -1.00770000   -2.27654600    0.73167300
 H                   1.90009000    0.73024900    1.14626600
```

Imaginary frequency= -421.9325 **cm-1**

TS 14-11

```
B3LYP/6-31G*; Gaussian 03, Revision B.05
Point group: C1
```

State= 1-A
HF= -302.2873097 Hartree
Zero-point correction= 0.076188 (Hartree/Particle)
Sum of electronic and zero-point Energies= -302.240733 Hartree

N	0.74834700	-1.31161400	0.00973900
N	-2.17926600	0.02442300	-0.42611800
C	-0.44092500	-1.20584300	0.41814300
C	1.61458100	-0.33001000	-0.26419800
C	1.20822300	1.00356100	-0.07990000
C	-0.07990900	1.35304300	0.25982200
C	-1.14586400	0.42363500	0.04398100
H	-0.32556800	2.31905700	0.68789600
H	-1.22909500	-1.94322700	0.40928900
H	1.97816800	1.76972200	-0.06155000
H	2.65629000	-0.60153300	-0.38806400

Imaginary frequency= -443.9761 **cm-1**

TS 16-11

B3LYP/6-31G*; Gaussian 03, Revision B.05
Point group: C1
State= 1-A
HF= -302.305887 Hartree
Zero-point correction= 0.076890 (Hartree/Particle)
Sum of electronic and zero-point Energies= -302.257905 Hartree

N	0.18031900	1.41253200	0.08603200
N	1.61080900	-0.87894200	-0.46709300
C	-0.77361700	-1.33097200	0.30862400
C	-1.55486800	-0.22515400	-0.03326800
C	-1.06105200	1.06745300	-0.28121000
C	1.31826400	1.03518300	0.28264700
C	0.56764000	-1.26143400	-0.03914000
H	-1.18433500	-2.20713800	0.79568800
H	-2.63870900	-0.33200200	-0.03452600
H	-1.70723200	1.87232400	-0.61039500
H	2.01418100	1.22122600	1.09074200

Imaginary frequency= -310.1164 **cm-1**

TS 10-17

B3LYP/6-31G*; Gaussian 03, Revision B.05
Point group: C1
State= 1-A
HF= -302.2780849 Hartree
Zero-point correction= 0.076870 (Hartree/Particle)
Sum of electronic and zero-point Energies= -302.230093 Hartree

N	-1.07582100	-1.13064500	-0.06812500
N	2.22208900	0.07089800	-0.58952000
C	1.19161500	-0.24465200	-0.06539600
C	0.13971300	-1.13467500	0.44367600
C	-1.61796500	0.01534900	-0.39880100

C	-0.92424800	1.20161700	0.01534800
C	0.30466900	1.01825400	0.56363300
H	0.37048000	-1.69835100	1.34483600
H	-2.55896300	0.02660600	-0.93955700
H	-1.38352000	2.18052400	-0.08359300
H	0.98542300	1.77409200	0.93106100

Imaginary frequency= -464.2446 **cm-1**

TS 11-18

RB3LYP/6-31G*; Gaussian 03, Revision B.05
Point group: C1
State= 1-A
HF= -302.2969919 Hartree
Zero-point correction= 0.076430 (Hartree/Particle)
Sum of electronic and zero-point Energies= -302.249952 Hartree

N	1.10338300	-1.12430800	-0.10413000
N	-2.30554100	-0.28865200	-0.54517600
C	0.16861100	-1.32195200	0.67265700
C	-1.42995200	0.32619900	-0.07238900
C	-0.36314400	1.06088400	0.51107600
C	0.92020800	1.17803200	-0.06377000
C	1.65546400	0.06127900	-0.43227300
H	-0.68071600	-1.99859600	0.55711000
H	-0.68543900	1.83896900	1.20068800
H	1.42130900	2.14292800	-0.05290200
H	2.65283600	0.08077400	-0.85155900

Imaginary frequency= -373.5933 **cm-1**

Calculated electronic transitions and simulated UV–vis spectra

TD-B3LYP/6-31G* calculations (first 10 excited states)

45

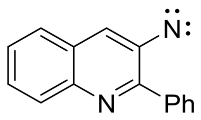

Symmetry Cs
State=3-A", S2= 2.057673
HF=-687.0504733

Zero-point correction= 0.207435
Sum of electronic and thermal Free Energies= -686.884407

N	0.48718000	-0.70727800	0.00000000
N	-2.04771200	1.91199500	0.00000000
C	-2.52429100	-2.90558100	0.00000000
C	-1.90777600	-4.13822200	0.00000000
C	-0.49522100	-4.23009800	0.00000000

C	0.28555600	-3.08834200	0.00000000
C	-0.31755500	-1.81175100	0.00000000
C	-1.74679600	-1.71461300	0.00000000
C	0.00000000	0.50969600	0.00000000
C	-1.47347600	0.73004500	0.00000000
C	-2.30172700	-0.42735700	0.00000000
C	0.97812600	1.62814100	0.00000000
C	2.35351000	1.31810100	0.00000000
C	3.31269600	2.32370100	0.00000000
C	2.92598200	3.66714200	0.00000000
C	1.56916600	3.98931400	0.00000000
C	0.60377900	2.98270600	0.00000000
H	-3.60852600	-2.82594600	0.00000000
H	-2.50533900	-5.04532800	0.00000000
H	-0.02278200	-5.20837400	0.00000000
H	1.36973300	-3.13891800	0.00000000
H	-3.37833000	-0.28486500	0.00000000
H	2.64637900	0.27482800	0.00000000
H	4.36700800	2.05973600	0.00000000
H	3.67600800	4.45371700	0.00000000
H	1.25437300	5.02930200	0.00000000
H	-0.44664300	3.25553000	0.00000000

Vibrational frequencies (scaled by 0.9613):

ModeNr.	Symmetry	Wavenumber	Abs.Int.	Rel.Int.
1	A"	13.6	0.2	0
2	A"	52.1	1.9	2
3	A"	100.0	0.1	0
4	A'	125.0	0.8	1
5	A"	155.7	0.0	0
6	A"	245.4	4.2	5
7	A'	271.4	0.2	0
8	A'	306.6	0.3	0
9	A"	309.8	0.7	1
10	A'	325.5	5.4	7
11	A"	395.3	0.0	0
12	A"	417.0	1.7	2
13	A'	440.4	2.2	3
14	A"	454.8	1.5	2
15	A"	490.8	7.6	10
16	A'	524.1	19.0	24
17	A"	537.9	2.0	2
18	A'	556.7	1.0	1
19	A'	594.2	5.0	6
20	A'	612.0	1.8	2
21	A'	666.3	2.8	4
22	A"	671.4	17.9	23
23	A"	695.7	18.4	23
24	A'	703.8	0.0	0
25	A"	731.6	11.8	15
26	A"	748.1	39.3	50
27	A"	775.5	5.9	7
28	A'	794.2	0.2	0
29	A"	830.0	1.6	2

30	A"	832.0	19.8	25
31	A"	856.6	6.9	9
32	A'	882.8	0.1	0
33	A"	912.8	2.2	3
34	A"	924.8	2.4	3
35	A"	946.6	0.1	0
36	A"	954.5	0.2	0
37	A'	959.8	17.7	22
38	A"	969.1	0.3	0
39	A'	978.3	0.8	1
40	A'	1005.0	2.1	3
41	A'	1021.3	2.3	3
42	A'	1076.3	6.7	8
43	A'	1108.4	3.9	5
44	A'	1130.9	2.0	3
45	A'	1147.4	1.8	2
46	A'	1158.6	23.9	30
47	A'	1171.9	19.0	24
48	A'	1191.1	15.0	19
49	A'	1221.8	8.2	10
50	A'	1234.4	11.6	15
51	A'	1263.2	28.4	36
52	A'	1300.0	42.5	54
53	A'	1314.9	13.3	17
54	A'	1317.0	8.5	11
55	A'	1336.6	6.2	8
56	A'	1363.5	15.5	20
57	A'	1423.4	29.3	37
58	A'	1436.2	7.8	10
59	A'	1462.1	7.2	9
60	A'	1480.4	2.9	4
61	A'	1513.3	79.0	100
62	A'	1527.9	22.3	28
63	A'	1571.0	1.0	1
64	A'	1586.3	12.6	16
65	A'	1594.1	2.0	3
66	A'	3059.9	0.7	1
67	A'	3063.4	2.3	3
68	A'	3068.9	13.8	17
69	A'	3073.7	8.9	11
70	A'	3076.9	18.0	23
71	A'	3084.8	3.2	4
72	A'	3085.8	38.5	49
73	A'	3086.8	22.3	28
74	A'	3098.0	14.0	18
75	A'	3112.3	4.8	6

```
Excited State   1:   Triplet-A'     2.2343 eV  554.92 nm  f=0.0116
Excited State   2:   Triplet-A'     2.7770 eV  446.47 nm  f=0.0014
Excited State   3:   Triplet-A'     2.8865 eV  429.52 nm  f=0.0767
Excited State   4:   Triplet-A'     2.9973 eV  413.64 nm  f=0.0225
Excited State   5:   Triplet-A"     3.0160 eV  411.08 nm  f=0.0001
Excited State   6:   Triplet-A"     3.2029 eV  387.09 nm  f=0.0000
Excited State   7:   Triplet-A'     3.2954 eV  376.23 nm  f=0.0558
```

```
Excited State   8:   Triplet-A"     3.4045 eV  364.17 nm  f=0.0002
Excited State   9:   Triplet-A'     3.5034 eV  353.90 nm  f=0.0045
Excited State  10:   Triplet-A"     3.6935 eV  335.68 nm  f=0.0000
```

46

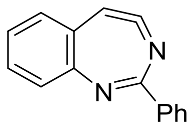


```
Symmetry C1
State 1-A
HF=-687.0497472

Zero-point correction=                           0.208392
Sum of electronic and thermal Free Energies=          -686.880606
```

N	-0.30750300	-0.60128500	0.00273300
N	0.23995200	1.58947300	-0.75920700
C	-3.86392700	0.61050600	0.35987500
C	-4.52554300	-0.55625100	0.00207100
C	-3.78866500	-1.67920100	-0.40057700
C	-2.40288900	-1.63353800	-0.40665200
C	-1.70238300	-0.46359900	-0.04405800
C	-2.46035400	0.68927700	0.33756300
C	0.59801100	0.27455400	-0.24004500
C	-0.65120800	2.04242900	-0.02251500
C	-1.73802700	1.91540600	0.72242600
C	2.03427500	-0.01512600	-0.08818000
C	2.45678600	-1.26458900	0.39718500
C	3.81306400	-1.53613300	0.54007000
C	4.76409000	-0.56782000	0.20069900
C	4.35063200	0.67462700	-0.28249000
C	2.99209200	0.95286000	-0.42561500
H	-4.42735500	1.48659100	0.67119200
H	-5.61062200	-0.59849400	0.03969200
H	-4.30056700	-2.59361900	-0.68721100
H	-1.81568100	-2.50429500	-0.68170200
H	-2.04085200	2.57914000	1.52219000
H	1.70644100	-2.00495400	0.65279800
H	4.13260700	-2.50409100	0.91654300
H	5.82339400	-0.78294600	0.31354300
H	5.08602800	1.42903400	-0.54809900
H	2.66374900	1.91590500	-0.80216300

Vibrational frequencies (scaled by 0.9613):

ModeNr.	Symmetry	Wavenumber	Abs.Int.	Rel.Int.
1	A	43.5	0.1	0

2	A	52.7	1.3	0
3	A	87.4	1.1	0
4	A	146.5	1.5	0
5	A	157.7	0.5	0
6	A	203.9	0.5	0
7	A	264.9	0.1	0
8	A	333.5	2.8	1
9	A	349.0	3.1	1
10	A	357.4	10.2	3
11	A	400.7	0.2	0
12	A	411.7	4.0	1
13	A	449.0	3.3	1
14	A	466.7	4.9	1
15	A	480.0	4.5	1
16	A	529.2	1.5	0
17	A	550.9	23.2	7
18	A	579.8	1.7	0
19	A	607.8	0.3	0
20	A	612.2	6.4	2
21	A	645.1	2.5	1
22	A	670.7	6.8	2
23	A	674.7	12.4	4
24	A	687.1	41.7	12
25	A	731.0	16.1	5
26	A	738.3	21.0	6
27	A	758.6	43.6	13
28	A	767.8	17.6	5
29	A	790.4	2.0	1
30	A	833.3	0.0	0
31	A	844.5	1.0	0
32	A	856.9	1.1	0
33	A	915.5	1.6	0
34	A	918.7	2.4	1
35	A	946.0	0.0	0
36	A	947.9	5.4	2
37	A	953.0	101.9	30
38	A	967.6	0.2	0
39	A	979.4	0.5	0
40	A	1016.9	1.2	0
41	A	1023.1	3.9	1
42	A	1069.1	5.9	2
43	A	1089.2	7.2	2
44	A	1102.8	17.3	5
45	A	1140.8	20.9	6
46	A	1147.6	0.8	0
47	A	1158.2	89.0	26
48	A	1167.4	20.6	6
49	A	1176.9	15.8	5
50	A	1223.4	8.9	3
51	A	1248.0	73.9	22
52	A	1278.6	0.7	0
53	A	1298.9	6.6	2
54	A	1315.5	5.4	2
55	A	1317.4	1.8	1
56	A	1434.7	0.4	0
57	A	1439.1	17.8	5

58	A	1444.8	28.3	8
59	A	1481.7	3.8	1
60	A	1538.2	3.8	1
61	A	1562.5	79.1	23
62	A	1575.0	1.7	1
63	A	1596.4	9.6	3
64	A	1606.5	57.6	17
65	A	1921.4	338.8	100
66	A	3060.6	2.2	1
67	A	3062.8	0.1	0
68	A	3070.4	10.5	3
69	A	3073.6	16.7	5
70	A	3082.3	33.4	10
71	A	3084.5	31.2	9
72	A	3092.5	18.6	5
73	A	3099.9	4.8	1
74	A	3105.0	4.4	1
75	A	3117.0	1.8	1

```
Excited State   1:   Singlet-A       3.0960 eV  400.47 nm  f=0.0266
Excited State   2:   Singlet-A       3.4947 eV  354.78 nm  f=0.0146
Excited State   3:   Singlet-A       3.8427 eV  322.65 nm  f=0.1410
Excited State   4:   Singlet-A       4.1250 eV  300.57 nm  f=0.0966
Excited State   5:   Singlet-A       4.3976 eV  281.94 nm  f=0.1189
Excited State   6:   Singlet-A       4.5528 eV  272.32 nm  f=0.0174
Excited State   7:   Singlet-A       4.7710 eV  259.87 nm  f=0.1445
Excited State   8:   Singlet-A       4.8618 eV  255.02 nm  f=0.1018
Excited State   9:   Singlet-A       4.9615 eV  249.89 nm  f=0.0019
Excited State  10:   Singlet-A       5.0026 eV  247.84 nm  f=0.1900
```

47

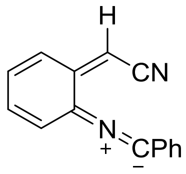

Symmetry Cs
State=1-A'
HF=-687.0294323

Zero-point correction= 0.205827
Sum of electronic and thermal Free Energies= -686.865771

N	-1.76695500	-2.26383600	0.00000000
N	0.00000000	0.62850200	0.00000000
C	-2.71687100	3.74139500	0.00000000
C	-1.31705500	4.00764200	0.00000000
C	-0.44485100	2.95188100	0.00000000
C	-0.92759800	1.61217600	0.00000000
C	-2.34253800	1.29717300	0.00000000

C	-3.19634500	2.46082900	0.00000000
C	-2.93056400	0.03265600	0.00000000
C	-2.27821600	-1.20823700	0.00000000
C	0.80768700	-0.21726500	0.00000000
C	1.80274500	-1.21360200	0.00000000
C	1.43739200	-2.57999600	0.00000000
C	2.44356500	-3.54037500	0.00000000
C	3.79126300	-3.17011400	0.00000000
C	4.14578200	-1.81614800	0.00000000
C	3.16583200	-0.83306000	0.00000000
H	-3.41733700	4.57255200	0.00000000
H	-0.94884000	5.02818700	0.00000000
H	0.63027500	3.10784500	0.00000000
H	-4.26851300	2.28392100	0.00000000
H	-4.01558900	-0.01288600	0.00000000
H	0.38590900	-2.85125700	0.00000000
H	2.16812900	-4.59097500	0.00000000
H	4.56496900	-3.93230200	0.00000000
H	5.19236500	-1.52573000	0.00000000
H	3.43595500	0.21825300	0.00000000

Vibrational frequencies (scaled by 0.9613):

ModeNr.	Symmetry	Wavenumber	Abs.Int.	Rel.Int.
1	A"	27.2	1.5	0
2	A"	44.2	0.5	0
3	A"	72.2	1.9	0
4	A'	76.5	0.8	0
5	A'	125.1	2.0	0
6	A"	139.0	0.0	0
7	A'	162.2	6.7	1
8	A"	201.3	0.6	0
9	A'	228.5	1.4	0
10	A"	271.3	1.6	0
11	A'	352.6	2.3	0
12	A"	361.2	0.1	0
13	A"	391.6	0.0	0
14	A'	423.6	0.9	0
15	A"	441.2	8.4	1
16	A"	471.9	4.9	1
17	A'	487.2	1.4	0
18	A"	496.0	3.8	1
19	A"	534.6	0.6	0
20	A'	539.1	24.2	4
21	A'	565.7	4.3	1
22	A'	611.9	0.3	0
23	A'	628.6	11.9	2
24	A"	634.7	9.1	2
25	A"	667.9	14.8	2
26	A'	671.1	5.1	1
27	A"	699.0	0.2	0
28	A"	722.2	63.4	10
29	A"	731.1	50.7	8
30	A'	773.6	11.3	2
31	A"	815.2	13.1	2

32	A"	825.6	0.7	0
33	A'	861.6	13.2	2
34	A"	893.3	0.3	0
35	A"	898.1	3.3	1
36	A"	942.5	0.3	0
37	A"	942.7	0.6	0
38	A'	968.4	2.3	0
39	A"	977.0	0.9	0
40	A'	994.5	11.9	2
41	A'	1013.1	2.5	0
42	A'	1040.1	7.0	1
43	A'	1076.9	4.1	1
44	A'	1084.2	56.7	9
45	A'	1133.8	60.5	10
46	A'	1147.2	13.0	2
47	A'	1151.3	0.8	0
48	A'	1175.7	3.5	1
49	A'	1197.7	18.5	3
50	A'	1234.0	42.4	7
51	A'	1282.3	27.5	5
52	A'	1290.3	79.3	13
53	A'	1326.2	2.4	0
54	A'	1359.5	9.1	2
55	A'	1379.3	63.1	10
56	A'	1430.0	4.7	1
57	A'	1447.7	74.5	12
58	A'	1472.3	24.2	4
59	A'	1493.5	200.2	33
60	A'	1505.6	100.0	17
61	A'	1551.1	1.7	0
62	A'	1581.6	37.7	6
63	A'	1604.6	100.2	17
64	A'	2172.0	202.5	34
65	A'	2234.0	604.4	100
66	A'	3061.1	5.2	1
67	A'	3068.0	0.3	0
68	A'	3070.6	1.6	0
69	A'	3071.8	2.2	0
70	A'	3078.1	40.7	7
71	A'	3078.6	7.1	1
72	A'	3085.4	8.5	1
73	A'	3090.5	10.3	2
74	A'	3095.9	33.3	6
75	A'	3096.3	17.0	3

```
Excited State   1:   Singlet-A'     2.1519 eV  576.17 nm  f=0.2607
Excited State   2:   Singlet-A"     2.4022 eV  516.12 nm  f=0.0000
Excited State   3:   Singlet-A'     3.2737 eV  378.72 nm  f=0.0024
Excited State   4:   Singlet-A'     3.6274 eV  341.80 nm  f=0.0081
Excited State   5:   Singlet-A'     4.0075 eV  309.38 nm  f=0.1594
Excited State   6:   Singlet-A'     4.3768 eV  283.28 nm  f=0.2323
Excited State   7:   Singlet-A"     4.6048 eV  269.25 nm  f=0.0000
Excited State   8:   Singlet-A"     4.6901 eV  264.35 nm  f=0.0000
Excited State   9:   Singlet-A'     4.7170 eV  262.84 nm  f=0.0233
Excited State  10:   Singlet-A'     4.8904 eV  253.52 nm  f=0.5817
```

49

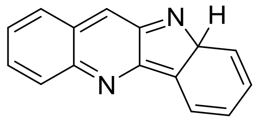

Symmetry C1
State=1-A
HF= -687.0544915

Zero-point correction= 0.209095
Sum of electronic and thermal Free Energies= -686.882840

C	3.53701300	-1.15102300	-0.12484900
C	4.53855600	-0.22701000	-0.15079500
C	4.24144200	1.17625400	-0.08773800
C	2.95206500	1.61340400	0.00091700
C	1.85387300	0.68762300	0.03464200
C	2.15716300	-0.75013300	-0.03215700
N	0.61842900	1.18102600	0.12853700
C	-0.38148600	0.27767600	0.14940400
C	-0.20889600	-1.18121400	0.08076700
C	1.12431900	-1.67056500	0.00447200
C	-1.76135800	0.49245800	0.20950600
C	-2.36307300	-0.87727100	0.29440800
N	-1.33235000	-1.87995900	0.09334100
C	-2.58813100	1.63464100	0.11776600
C	-3.92722300	1.45453700	-0.13749900
C	-4.49438900	0.14290800	-0.34915600
C	-3.75268300	-0.98827100	-0.23235000
H	3.75847700	-2.21443700	-0.17347200
H	5.57465200	-0.54670000	-0.22042700
H	5.05957200	1.89095000	-0.11126500
H	2.70458300	2.66916000	0.05020600
H	1.31609000	-2.73962100	-0.03554300
H	-2.54405800	-1.01623100	1.39199400
H	-2.14937700	2.62738600	0.15957500
H	-4.57566300	2.31991700	-0.24421100
H	-5.53945100	0.07556800	-0.64031800
H	-4.17052900	-1.97754500	-0.39371900

Vibrational frequencies (scaled by 0.9613):

ModeNr.	Symmetry	Wavenumber	Abs.Int.	Rel.Int.
1	A	62.6	2.0	3
2	A	95.8	0.3	1
3	A	148.5	2.5	4
4	A	155.0	2.3	4
5	A	195.9	0.7	1
6	A	286.4	2.8	5
7	A	298.4	4.0	7
8	A	314.3	3.6	6
9	A	358.5	0.9	1
10	A	400.4	5.7	9
11	A	450.9	7.2	12
12	A	464.8	13.6	22
13	A	469.3	25.3	41
14	A	506.9	7.1	11

15	A	524.3	0.8	1
16	A	532.2	1.3	2
17	A	556.6	2.2	3
18	A	587.9	1.3	2
19	A	602.6	7.8	13
20	A	669.3	3.8	6
21	A	681.4	38.8	63
22	A	704.0	15.9	26
23	A	726.4	7.3	12
24	A	735.2	37.1	60
25	A	750.8	40.2	65
26	A	757.7	1.9	3
27	A	772.5	4.3	7
28	A	811.3	0.6	1
29	A	835.5	8.1	13
30	A	836.4	2.4	4
31	A	879.5	18.2	29
32	A	884.1	2.1	3
33	A	928.9	1.0	2
34	A	935.8	1.8	3
35	A	943.6	14.6	24
36	A	953.2	13.8	22
37	A	958.7	14.2	23
38	A	961.1	0.2	0
39	A	981.2	6.1	10
40	A	988.1	29.4	47
41	A	1018.8	23.6	38
42	A	1079.1	4.3	7
43	A	1109.1	30.4	49
44	A	1121.5	7.2	12
45	A	1134.6	8.5	14
46	A	1146.5	1.9	3
47	A	1160.1	4.9	8
48	A	1174.7	0.1	0
49	A	1213.5	10.7	17
50	A	1236.8	1.8	3
51	A	1302.1	33.9	55
52	A	1316.7	7.5	12
53	A	1325.8	9.9	16
54	A	1352.8	25.2	41
55	A	1365.3	3.9	6
56	A	1394.9	2.5	4
57	A	1405.1	15.6	25
58	A	1441.5	8.9	14
59	A	1468.4	10.4	17
60	A	1488.3	40.5	65
61	A	1516.0	62.4	101
62	A	1530.0	13.3	21
63	A	1564.7	21.3	34
64	A	1602.7	12.0	19
65	A	1624.0	8.1	13
66	A	2725.6	12.1	19
67	A	3059.2	0.6	1
68	A	3063.4	10.7	17
69	A	3070.0	11.7	19
70	A	3075.2	6.9	11
71	A	3076.8	8.3	13
72	A	3083.4	35.2	57
73	A	3088.6	15.3	25
74	A	3093.3	26.1	42
75	A	3095.9	20.9	34

```
Excited State   1:   Singlet-A      2.0276 eV  611.48 nm  f=0.1358
Excited State   2:   Singlet-A      2.6689 eV  464.56 nm  f=0.0049
Excited State   3:   Singlet-A      2.8787 eV  430.70 nm  f=0.0421
Excited State   4:   Singlet-A      3.3600 eV  369.00 nm  f=0.1485
Excited State   5:   Singlet-A      3.6789 eV  337.01 nm  f=0.0627
Excited State   6:   Singlet-A      3.8956 eV  318.27 nm  f=0.0150
Excited State   7:   Singlet-A      4.2184 eV  293.91 nm  f=0.0128
Excited State   8:   Singlet-A      4.3211 eV  286.93 nm  f=0.0015
Excited State   9:   Singlet-A      4.5429 eV  272.92 nm  f=0.0060
Excited State  10:   Singlet-A      4.6564 eV  266.27 nm  f=0.0911
```

References

[1] Gaussian 03, Revision C.05, Frisch, M. J.; Trucks, G. W.; Schlegel, H. B.; Scuseria, G. E.; Robb, M. A.; Cheeseman, J. R.; Montgomery, Jr., J. A.; Vreven, T.; Kudin, K. N.; Burant, J. C.; Millam, J. M.; Iyengar, S. S.; Tomasi, J.; Barone, V.; Mennucci, B.; Cossi, M.; Scalmani, G.; Rega, N.; Petersson, G. A.; Nakatsuji, H.; Hada, M.; Ehara, M.; Toyota, K.; Fukuda, R.; Hasegawa, J.; Ishida, M.; Nakajima, T.; Honda, Y.; Kitao, O.; Nakai, H.; Klene, M.; Li, X.; Knox, J. E.; Hratchian, H. P.; Cross, J. B.; Bakken, V.; Adamo, C.; Jaramillo, J.; Gomperts, R.; Stratmann, R. E.; Yazyev, O.; Austin, A. J.; Cammi, R.; Pomelli, C.; Ochterski, J. W.; Ayala, P. Y.; Morokuma, K.; Voth, G. A.; Salvador, P.; Dannenberg, J. J.; Zakrzewski, V. G.; Dapprich, S.; Daniels, A. D.; Strain, M. C.; Farkas, O.; Malick, D. K.; Rabuck, A. D.; Raghavachari, K.; Foresman, J. B.; Ortiz, J. V.; Cui, Q.; Baboul, A. G.; Clifford, S.; Cioslowski, J.; Stefanov, B. B.; Liu, G.; Liashenko, A.; Piskorz, P.; Komaromi, I.; Martin, R. L.; Fox, D. J.; Keith, T.; Al-Laham, M. A.; Peng, C. Y.; Nanayakkara, A.; Challacombe, M.; Gill, P. M. W.; Johnson, B.; Chen, W.; Wong, M. W.; Gonzalez, C.; and Pople, J. A.; Gaussian, Inc., Wallingford CT, 2004.

[2] Wong, M. W. *Chem. Phys. Lett.* **1996**, *256*, 16502.

[3] Johnson, W. T. G.; Sullivan, M. B.; Cramer, C. J. *Int. J. Quantum. Chem.* **2001**, *85*, 492.

[4] Maltsev, A.; Bally, T.; Tsao, M.-L.; Platz, M. S.; Kuhn, A.; Vosswinkel, M.; Wentrup, C. *J. Am. Chem. Soc.* **2004**, *126*, 237.
